# Defining functional interactions during biogenesis of epithelial junctions

**DOI:** 10.1038/ncomms13542

**Published:** 2016-12-06

**Authors:** J. C. Erasmus, S. Bruche, L. Pizarro, N. Maimari, T. Pogglioli, C. Tomlinson, J. Lees, I. Zalivina, A. Wheeler, A. Alberts, A. Russo, V. M. M. Braga

**Affiliations:** 1 National Heart and Lung Institute, Faculty of Medicine, Imperial College London, London SW7 2AZ, UK; 2 Computing Department, Imperial College London, London SW7 2AZ, UK; 3 Bioengineering Department, Faculty of Engineering, Imperial College London, London SW7 2AZ, UK; 4 Department of Surgery & Cancer, Faculty of Medicine, Imperial College London, London SW7 2AZ, UK; 5 Department Structural and Molecular Biology, University College London, London WC1E 6BT, UK; 6 Van Andel Institute, Grand Rapids, Michigan 49503, USA

## Abstract

In spite of extensive recent progress, a comprehensive understanding of how actin cytoskeleton remodelling supports stable junctions remains to be established. Here we design a platform that integrates actin functions with optimized phenotypic clustering and identify new cytoskeletal proteins, their functional hierarchy and pathways that modulate E-cadherin adhesion. Depletion of EEF1A, an actin bundling protein, increases E-cadherin levels at junctions without a corresponding reinforcement of cell–cell contacts. This unexpected result reflects a more dynamic and mobile junctional actin in EEF1A-depleted cells. A partner for EEF1A in cadherin contact maintenance is the formin DIAPH2, which interacts with EEF1A. In contrast, depletion of either the endocytic regulator TRIP10 or the Rho GTPase activator VAV2 reduces E-cadherin levels at junctions. TRIP10 binds to and requires VAV2 function for its junctional localization. Overall, we present new conceptual insights on junction stabilization, which integrate known and novel pathways with impact for epithelial morphogenesis, homeostasis and diseases.

## Abstract (web summary)

Formation and reinforcement of E-cadherin-mediated adhesion depends on intracellular trafficking and interactions with the actin cytoskeleton, but how these are coordinated is not known. Here the authors conduct a focused phenotypic screen to identify new pathways regulating cell–cell junction homeostasis.

Multi-cellularity and adaptation to distinct environments underlie the evolutionary success of metazoans^1^,^2^. A key aspect of multi-cellularity is cell–cell adhesion, which ultimately allowed specialization of different tissues to cooperate and perform distinct tasks. E-cadherin, the first described member of the cadherin super-family, is essential for embryonic survival^3^ and a paradigm for cell–cell adhesion regulation. Dynamic cell–cell contacts are required for tissue integrity and morphogenesis, including cell division, epithelial sheet folding, lumen formation and geometric cell shape in different organisms^4^. Conversely, dysfunction of E-cadherin adhesion leads to the inability of cells to attach strongly to each other, sustain stress or maintain epithelial cell shape, thereby compromising tissue function in different diseases^5^,^6^.

Formation of E-cadherin-mediated adhesion is a complex, multi-layered process, involving engagement of receptors in neighbouring cells, reinforcement of attachment and reorganization of the underlying cortical cytoskeleton. Reinforcement of cadherin adhesion is achieved by intracellular trafficking (receptor delivery and turnover at the cell surface) and an indirect association with actin filaments^3^,^4^, which drives tension alongside contacts^7^. It is unclear how these different cellular processes are coordinated at the molecular level or how clustered receptors are kept in place to reinforce junctions^5^,^8^.

It is likely that the epithelial actin organization is maintained by integration of distinct subsets of actin remodelling properties. Distinct actin proteins act to polymerize, cap, sever, branch, cross-link and bundle filaments, and contribute to the reorganization of actin cytoskeleton as distinct cellular structures. In epithelia, polymerization and contraction have well-established roles in cadherin adhesion and morphogenetic processes^5^,^6^. Yet, how additional cytoskeletal proteins and actin remodelling functions collaborate during junction biogenesis is unclear.

Phenotypic analysis of RNAi screens^9^ have been instrumental in deciphering novel regulators of actin cytoskeleton^10^, cell shape^11^, cell motility^12^,^13^, or integrin-dependent adhesion and mechanosensing^14^,^15^ among others. Yet, while studies on invertebrate cell adhesion^16^, epithelial scattering^17^, or interactome of junctional proteins^18^ have been very informative, global understanding of cytoskeletal proteins required for junction formation has not been previously addressed. Nor is it clear how their function is integrated towards maintaining stable cadherin adhesion. In spite of the importance that junction dynamics have for many processes, large scale studies to investigate mechanisms and regulators of cell–cell contacts have been delayed by the absence of quantitative tools specific for epithelial structures and junctional markers. Here we set out to optimize methodology relevant for epithelial structures to identify new regulators and pathways required for junction stabilization.

We hypothesize that a similar phenotype upon depletion of specific cytoskeletal proteins may indicate cooperation to organize/maintain specific actin structures and stabilize cadherin receptors at junctions. Using optimized phenotypic clustering and enrichment analysis, we identify distinct functional profiles and actin-binding proteins that selectively perturb E-cadherin and junctional actin levels at newly formed contacts. Our computational analyses (i) associate a selective cytoskeletal protein network with specific biological output, (ii) allow interrogation of novel signalling pathways relevant for epithelial function and (iii) identify two new pathways that modulate E-cadherin adhesion. Further dissecting the functional hierarchy of cytoskeletal proteins in the context of cadherin adhesion will provide insights to overcome junction destabilization that occurs in tumour progression and a variety of epithelial pathologies.

## Results

### RNAi screen

**Figure 1:**
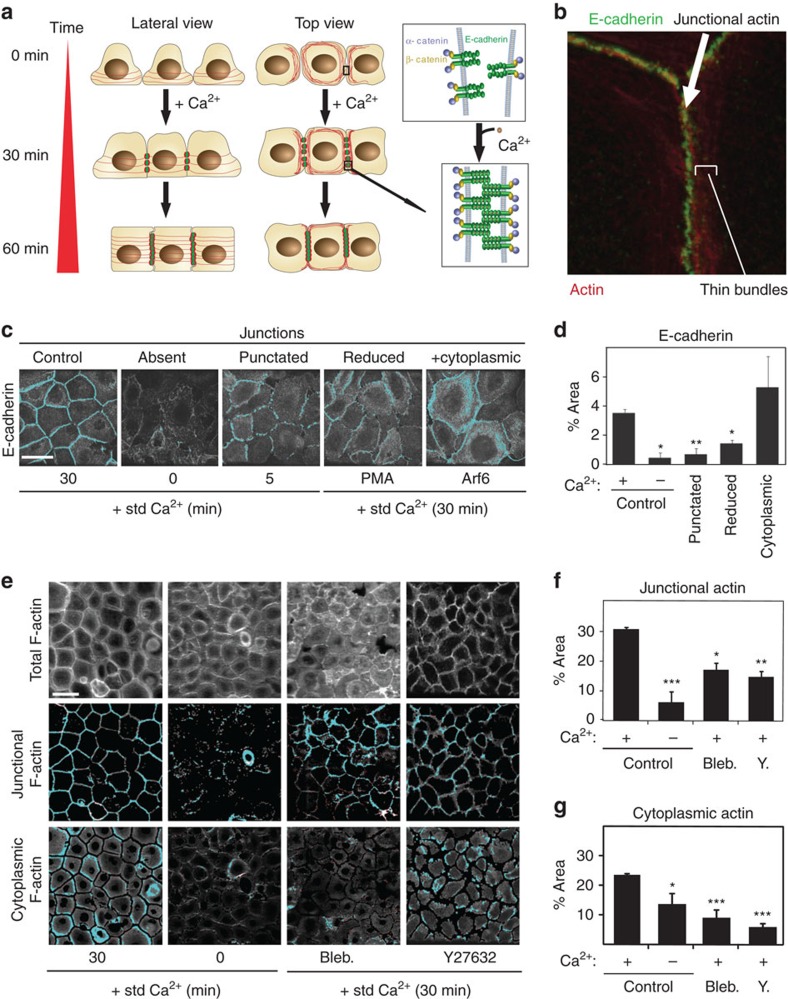
Automated analysis for quantification of epithelia-specific parameters. (**a**) Cell morphology changes during cell−cell adhesion. Diagrams are shown as lateral view (left), top view (middle) or representation of homophilic binding of E-cadherin (right). Addition of calcium ions induces assembly of E-cadherin puncta at the interface between neighbouring cells. F-actin re-organization is shown as appearance of junctional actin (co-localizes with E-cadherin) and circumferential thin bundles that compact towards junctions. (**b**) Immunofluorescence image showing E-cadherin localization at newly formed junctions (30 min; green), junctional actin and adjacent thin bundles (red). (**c**–**g**) Validation of automated image analysis (Supplementary Fig. 1). Positive and negative controls are cells induced to form junctions with standard calcium medium (30 min std.Ca^2+^) or maintained without cell−cell contacts (0 min std.Ca^2+^), respectively. (**c**) Keratinocytes were treated to generate images with E-cadherin staining as punctate (5 min std.Ca^2+^), reduced (PMA treatment) or increased cytoplasmic staining (active Arf6 expression). (**d**) Quantification of E-cad parameter as % thresholded area. (**e**) To validate image segmentation of F-actin pools, keratinocytes were pre-treated with blebbistatin (Bleb.) or Y27632 and junctions induced for 30 min in the presence of the drugs. (**f**,**g**) Images were quantified to generate the parameter Jun-A (F-actin pool co-localizing with E-cadherin at junctions, (**f**) or Cyt-A (that is, F-actin pool outside junctions and nucleus, (**g**). Data from three independent replicates; error bars represent standard error of the means. Scale bars, 25 μm. Statistical analysis *t*-test paired two samples, assuming equal variance; *P<0.02; **P<0.003; ***P<0.0004.

In spite of extensive progress in image analysis and the availability of increasingly sophisticated tools^10^,^17^,^19^,^20^, automated segmentation and quantification of junctional proteins in a confluent epithelial sheet have been challenging^21^,^22^,^23^. In particular, algorithms have not yet been established to segment F-actin at junctions and thin bundles (Fig. 1a,b), parameters with well-established biological relevance for epithelial junctions. To quantitatively monitor the effectiveness of cell–cell adhesion assembly, we developed a workflow (Supplementary Fig. 1, Fig. 1c–g) to quantify the levels of E-cadherin at junctions, junctional actin and peripheral thin bundles (Fig. 1a,b). A parameter for quality control of cell confluence was also validated to automatically eliminate images containing gaps in the monolayer (likely to result in false-negative data, Supplementary Fig. 2).

E-cadherin levels at junctions were detected by thresholding to minimize the contribution of the pool of receptors found in the cytoplasm (Fig. 1c). By comparing images containing different levels of cadherin at junctions, the percentage of pixels found in the thresholded image (% area) reliably detected partial disruption of E-cadherin levels and provided good dynamic range between negative and positive controls (Fig. 1c,d). This suggested that the segmentation successfully detected partial phenotypes obtained by RNAi. The parameters junctional actin (Jun-A) and cytoplasmic actin (Cyt-A) were successfully segmented (Supplementary Fig. 1) and quantified with good dynamic range to show disruption of the cytoskeleton as partial and distinct phenotypes (Fig. 1c–g). Thus, three parameters were validated to specifically detect quantitative changes in E-cadherin and cytoskeletal organization. The low dimensionality provided by parameters E-cad Jun-A and Cyt-A was considered against the inclusion of general biophysical features such as cell area, perimeter and so on. These biophysical features are not relevant for junction biogenesis *per se* nor add mechanistically to junction regulation; rather they may generate noise in subsequent phenotypic analysis.

**Figure 2:**
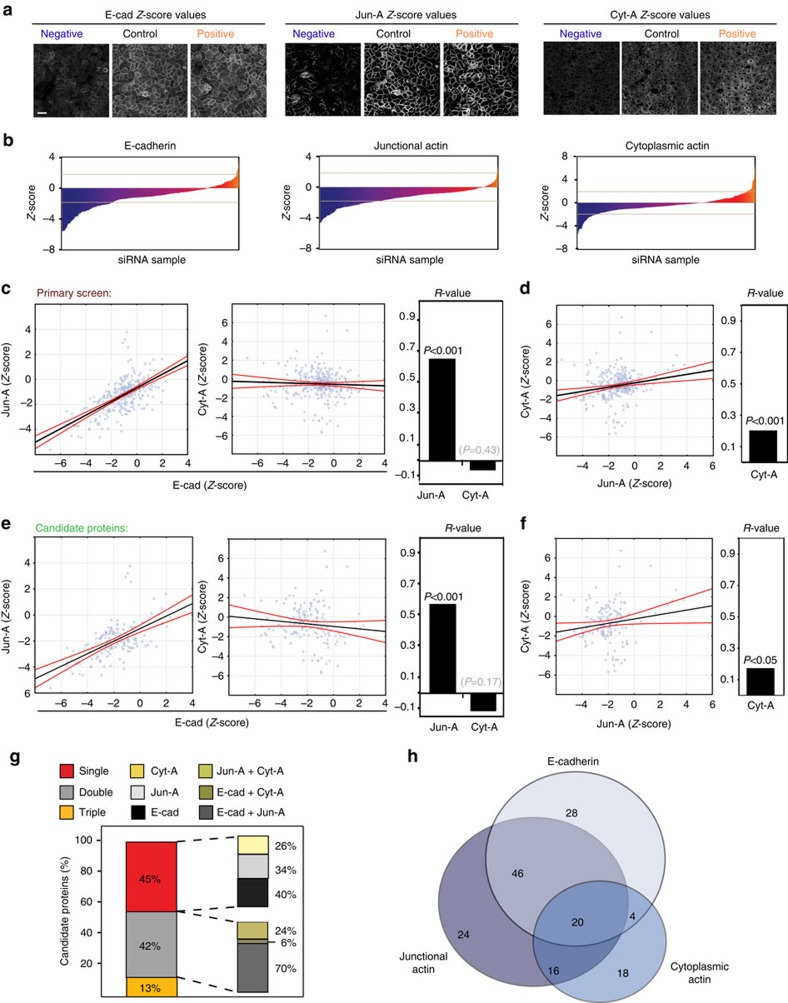
Global analysis of RNAi screen. (**a**) Representative processed images of the parameters E-cad, Jun-A and Cyt-A from controls and selected RNAi samples with positive or negative *Z*-scores. (**b**) Distribution of *Z*-scores of the primary RNAi screen data set (Supplementary Fig. 3a). Grey line marks cutoff *Z*-scores (+/− 1.65). (**c**–**f**) Correlation of the *Z*-scores of different parameters obtained in the primary screen (**c**,**d**) or the candidate proteins data sets (**e**,**f**) and their respective *R*-values. Partial interdependence of the parameters E-cad and Jun-A is observed, but not Cyt-A. Black lines represent the linear regression models and curved red lines show their 95% confidence interval. (**g**) Distribution of candidate proteins according to their phenotype showing interference with a single (single) or multiple parameters (double, triple) following depletion of a single protein. Values show the relative percentage in each subgroup. (**h**) Venn diagram showing the number of proteins and their corresponding phenotypes (single or overlapping phenotypes). Scale bar, 50 μm. Data represent median *Z*-score values of three independent replicates for each RNAi oligo. The number of data points (thereafter *N*=) for **b**–**d**, 327 or **e**–**h**, 156.

Confluent normal keratinocytes grown in the absence of cell–cell contacts were treated with the Actinome library^10^ (primary screen targeting 327 actin-binding proteins) (Supplementary Fig. 3a), junctions were induced for 30 min and images processed (Fig. 2a,b). The data set obtained did not follow Gaussian or log-Gaussian distribution (Supplementary Fig. 3b). To normalize between plates, *Z*-scores were calculated based on controls in each plate (see methods)^24^.

Strong effects of depletion of a given protein could result from a general perturbation of the actin cytoskeleton upon RNAi of core actin regulators. However, for the majority of samples we think that this is unlikely. First, overall total levels of F-actin in each sample did not correlate with the *Z*-scores obtained for E-cad or Jun-A (total F-actin intensity; Supplementary Fig. 3c,d), rather there was a stronger correlation with Cyt-A parameter (higher *R*-values). Second, E-cad, Jun-A or Cyt-A values did not correlate with cell number (nuclei count) or the presence of small gaps in the monolayer (% area total F-actin; Supplementary Fig. 4). Finally, a strong correlation between E-cad and Jun-A *Z*-scores (Fig. 2c) was consistent with their functional interdependence during cadherin assembly. In contrast, Cyt-A *Z*-scores values varied independently of either E-cad or Jun-A (Fig. 2c). Thus, the parameters measured behave as predicted from our current knowledge of regulation of E-cadherin adhesion. Taken together, our results suggest that *Z*-score variations by depletion of a specific cytoskeletal protein reflect a *bona fide* effect on E-cadherin adhesion and/or actin remodelling.

Based on *Z*-scores values, a subset of candidate proteins (156 proteins; Supplementary Table 1) was selected (Fig. 2b). Similar *Z*-score correlations as the primary screen were observed for this subset (Fig. 2e,f; Supplementary Fig. 3c,d), indicating that candidate proteins were a representative pool of the original data set. Overall, individual candidate proteins displayed phenotypes affecting mostly one or two parameters; Fig. 2g,h). Importantly, around 30 candidate proteins have been previously shown to regulate cadherin adhesion (Supplementary Table 2), among these there are regulators of actin (WAS, WASF1/2, VASP, ENAH, ABI, CYFP1), ERM proteins (RDX, EZR) and others (TRIOBP, MYOIV, FILA, FILB, EPB1L5 and so on.). This analysis supports and validates the methodology deployed here to identify both known and unknown junction regulators. In addition, some proteins have been shown to regulate epithelial differentiation, morphogenesis or participate in diseases (Supplementary Table 3). Clearly, the role of these proteins may be multi-factorial and, with a few exceptions, a direct regulation of cadherin adhesion in such pathologies (Supplementary Table 3) has not yet been formally demonstrated. Nevertheless, our data suggest that the new function on junctions identified herein could potentially contribute to defective morphogenesis or diseases in which candidate proteins have been implicated.

### Phenotypic clustering of candidate proteins

While identifying novel junction regulators per se is exciting, a major conceptual advance is to elucidate how specific proteins with similar junctional phenotypes cooperate to stabilize cell–cell adhesion. Such analysis would accelerate pathway inference and identification of regulatory mechanisms of the epithelial cytoskeleton. A phenotypic similarity analysis^25^ of candidate proteins (Supplementary Table 1) was optimized and validated statistically (Silhouette plots, Supplementary Fig. 5) and by functional enrichment (Supplementary Fig. 6a–d; see methods). Using these criteria, Spectral clustering^26^ using Mahalanobis distance was the best strategy for phenotypic clustering for our data set.

**Figure 3:**
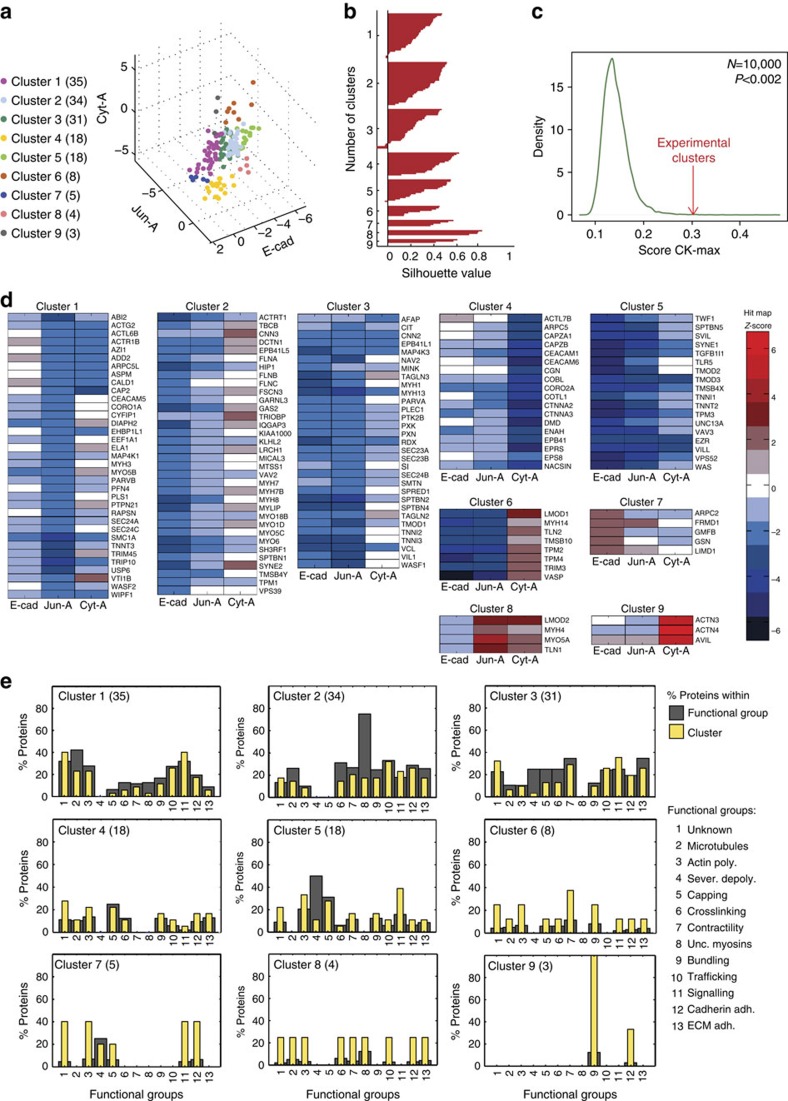
Phenotypic clustering. Using spectral clustering with Mahalanobis distance (Supplementary Figs 5 and 6), candidate proteins were grouped into nine separate clusters. (**a**) Distribution of clusters in three-dimensional space according to *Z*-scores for the parameters E-cad, Jun-A or Cyt-A. The number of proteins classified in each cluster is shown in parenthesis. (**b**) Silhouette plot shows a silhouette value (−1 to +1) for each point in the data set measuring how similar it is to points within its own cluster; positive values mean correct classification in its cluster. (**c**) Adequacy of experimental clusters based on their protein interaction landscape. Around 10,000 random clusters were plotted according to their Commuter time Kernel maximal score (CK-max) to produce the background curve of random clusters (green line). Red arrow points to where experimental clusters are found. (**d**) Heat map of the optimized clusters based on their corresponding *Z*-scores for E-cad, Jun-A and Cyt-A. Name of protein depleted is shown on the right of each panel. Data in **a**–**d** represent median *Z*-score values of three independent replicates for each RNAi oligo. (**e**) Functional enrichment. Following manual curation of the literature, each of the candidate proteins was attributed one or more functions on actin remodelling (functional group; *x* axis). Enrichment of the 13 functions in each cluster is shown in two ways: the distribution of actin functions relative to all functions found in the candidate protein data set (grey bars, that is, all proteins that cap, sever and so on.) or in the subset of functions found among proteins in the cluster (yellow bars). *N*=156.

Following further optimization (Supplementary Fig. 6e), the clusters projected in three-dimensional space showed partial overlap for clusters 1–3 (Fig. 3a) and very few data points were wrongly classified in their respective clusters (5 out of 156 samples with negative Silhouette values, Fig. 3b). To validate the optimized clusters, three additional approaches demonstrated their biological significance. First, proteins that participate in the same pathway/cellular function are likely to share binding partners and thus are found together in protein–protein interaction (PPI) networks (see methods)^27^. Based on neighbour interacting partners of individual candidate proteins, randomized clusters containing the same number of proteins of each phenotypic cluster were produced (Fig. 3c). The random clusters generated with the same composition as the phenotypic clusters (red arrow) were found outside the random background of the density distribution curve (peak, green line, Fig. 3c). This result suggests that the overall partition of the data set into nine clusters was highly significant. Thus, the proteins in the experimental clusters tend to be closer together in the protein interaction space than would be expected by chance.

Second, specific cellular phenotypes were well segregated among the different clusters (Fig. 3d). A spectrum of disruption of E-cadherin adhesion was observed across phenotypic clusters, from mild (that is, Cluster 2) to severe perturbation (that is, Cluster 5). In addition, depletion of proteins found in Cluster 1 mostly reduced F-actin at junctions (Jun-A), while a decrease in E-cadherin levels was predominant in Cluster 2. Clearly, in spite of the correlation between E-cad and Jun-A parameters (*R*-value 0.65; Fig. 2c,d), depletion of specific proteins interfered differentially with the levels of cadherin receptors or F-actin at junctions.

Third, as a specific phenotype profile is found in each cluster (for example, strong interference with cadherin levels), an enrichment of actin remodelling functions that are relevant to that phenotypic profile is predicted. However, there was no significant enrichment of annotated functions using GO classification, in contrast to previous RNAi screens^10^,^13^,^14^,^15^. Using manual curation of the literature, all published actin remodelling functions of each candidate protein were attributed in 13 functional groups (that is, polymerization, bundling and so on.). For each cluster (Fig. 3e), functional enrichment is shown in two ways: (i) as the percentage of all proteins allocated to a specific functional group (grey bars; that is, all capping proteins) and (ii) how enriched a particular remodelling function is among all proteins in a given cluster (yellow bars). Indeed, a striking concentration of specific functions was observed in Cluster 2 (unconventional myosins), while most proteins that sever/depolymerize F-actin accumulated in Clusters 3 and 5 (Fig. 3e).

Taken together, the above results on our data set indicate that the optimized clustering analysis is highly meaningful from the point of view of the interaction landscape, functional enrichment and biological phenotype on epithelial junctions. The systematic methodology optimization shown here can thus be a powerful approach to generate a framework and facilitate pathway discovery among the candidate proteins.

### Functional enrichment analysis

While distinct clusters can be obtained by phenotypic analysis^28^, the challenge is to find meaningful intra- and inter-clusters relationships that correlate imaging data with biological functions. We hypothesized that enrichment of distinct actin remodelling events in each cluster would allow predictions of cytoskeletal functions that differentially contribute to the formation of stable cadherin contacts. Overall, the identification of important actin remodelling for junction stabilization provides insights on the relationship between specific cytoskeletal processes and the extent of E-cadherin adhesion perturbation. For example, the stronger reduction of E-cadherin levels at junctions in cluster 5–6 highlights the overlooked contribution of actin filament turnover/stabilization for E-cadherin levels at cell–cell contacts.

**Figure 4:**
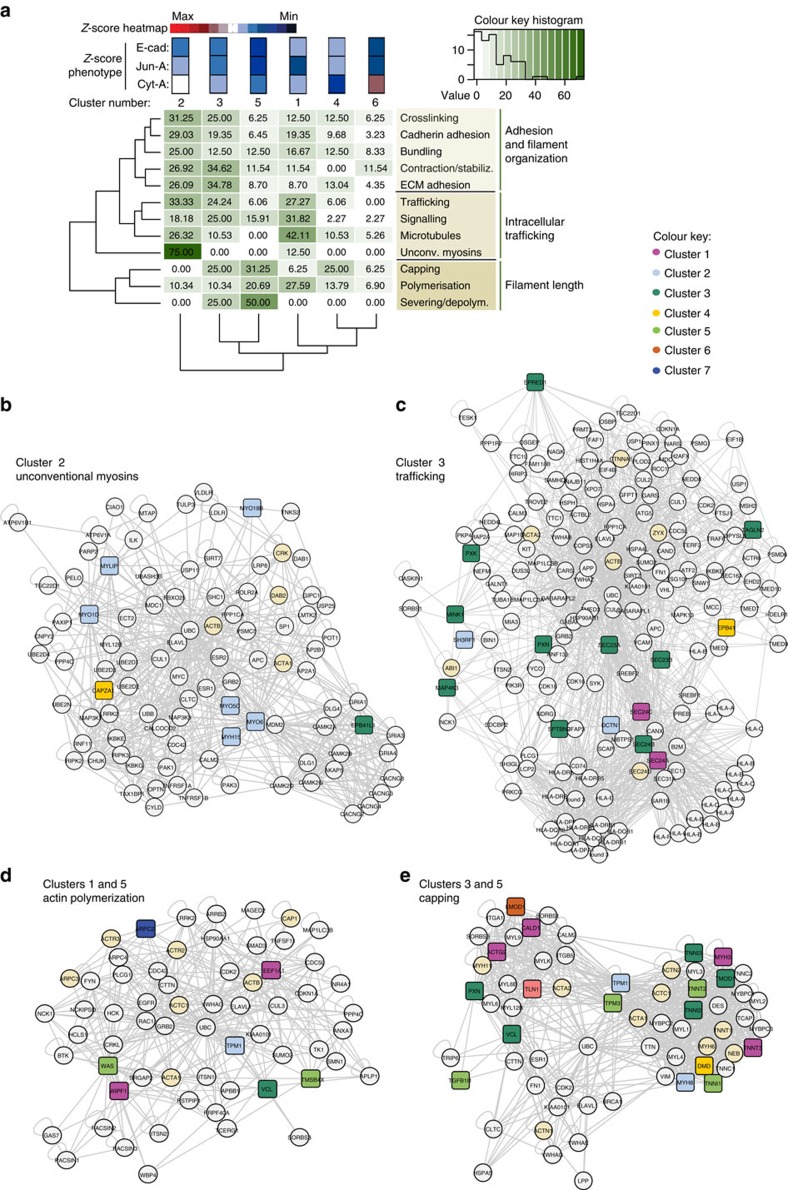
Protein–protein interaction networks show connectivity within and among different phenotypic clusters. (**a**) Hierarchical clustering of actin functions and phenotypic clusters. The enrichment of actin functions in specific clusters (as in Fig. 3e) is shown as a heat map. Protein functions are shown on the right and cluster number and phenotype are shown on the top. Analysis shows similarities among clusters containing specific profiles of functions along the grouping of functions controlling filament organization (top group on the right), intracellular trafficking (middle group on the right) or filament length (bottom group on the right). (**b**–**e**) Protein–protein interaction networks of unconventional myosins enriched in Cluster 2 (**b**), intracellular trafficking proteins in Cluster 3 (**c**), actin polymerization proteins found in Clusters 1 and 5 networks (**d**) or capping proteins present in Clusters 3 and 5 interaction maps (**e**). Clusters are colour-coded as shown in the colour key diagram. Grey circles represent proteins found in available PPI datasets.

Such insights are highlighted by the hierarchical clustering of phenotypic groups (based on *Z*-scores and function, Fig. 4a). Specific actin remodelling functions were enriched among clusters and classified in three main groups: control of adhesion and filament organization (Clusters 2, 3), intracellular trafficking (Clusters 1–3) or filament length (Clusters 1, 3–5). Clusters 1–3 contained the majority of proteins known to regulate cadherin adhesion (68%) and intracellular trafficking (85%, Fig. 4a). Considering the functional enrichment and the spectrum of E-cadherin disruption, milder phenotypes on E-cadherin adhesion correlated well with depletion of trafficking proteins (Clusters 1, 2) and stronger phenotypes correlated with de-regulation of filament length (Clusters 3, 5).

The hierarchical distribution of enriched functions would suggest a potential integration among the subsets of proteins in each cluster. In other words, can partners be found that interact with and modulate a specific enriched actin function to control a distinct junctional phenotype (that is, mild to severe defects in different clusters)? A high degree of PPIs for a specific functional property is observed either within a cluster (for example, Fig. 4b) or spanning different clusters; for example, trafficking (Fig. 4c, cluster 3), actin polymerization (Fig. 4d, common proteins in clusters 1 and 5) or capping (Fig. 4e, clusters 3 and 5). These results suggest that the actin functions of proteins found in distinct clusters may be coordinated to modulate a particular cellular event during cadherin adhesion. The implication is that, in our model system, distinct nodes of a pathway that stabilize junctions may also be found in separate phenotypic clusters.

### Novel regulators of E-cadherin adhesion

**Figure 5:**
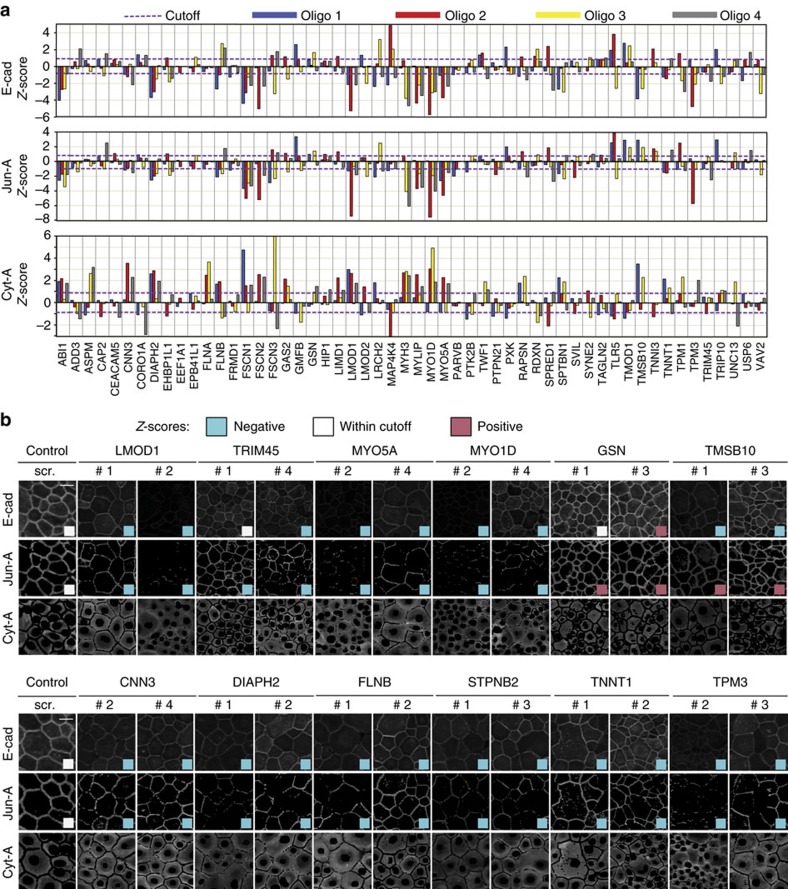
Validation of candidate proteins. The RNAi phenotype was re-tested in a selection of candidate proteins using four separate siRNA oligos for each protein. (**a**) Graphs showing the parameters E-Cad (top graph), Jun-A (middle graph) and Cyt-A (bottom graph). Median *Z*-score values were obtained from three independent replicates for each siRNA oligo. Proteins with two or more oligos with *Z*-scores outside cutoff were identified as validated proteins. (**b**) Representative processed images are shown of cells treated with controls (scrambled, scr.) or two selected siRNA oligos against proteins identified as validated proteins. Although only E-cad and Jun-A were used for selection, Cyt-A images are also shown for completeness. Coloured squares within images shows *Z*-scores below (blue), within (white) or above (pink) the cutoff. Scale bar, 50 μm.

To test the above predictions, we selected candidate proteins for validation with four individual oligos (see methods; Supplementary Fig. 3a) and plotted the median *Z*-scores from three experiments (Fig. 5a). Out of 49 proteins, 31 samples showed similar E-cad phenotypes with at least two independent oligos (thereafter referred as validated; Fig. 5b). Samples with ambiguous *Z*-scores (that is, two positive and two negative) were not considered and require further investigation. Overall, a number of new and known regulators of cadherin adhesion were validated.

**Figure 6:**
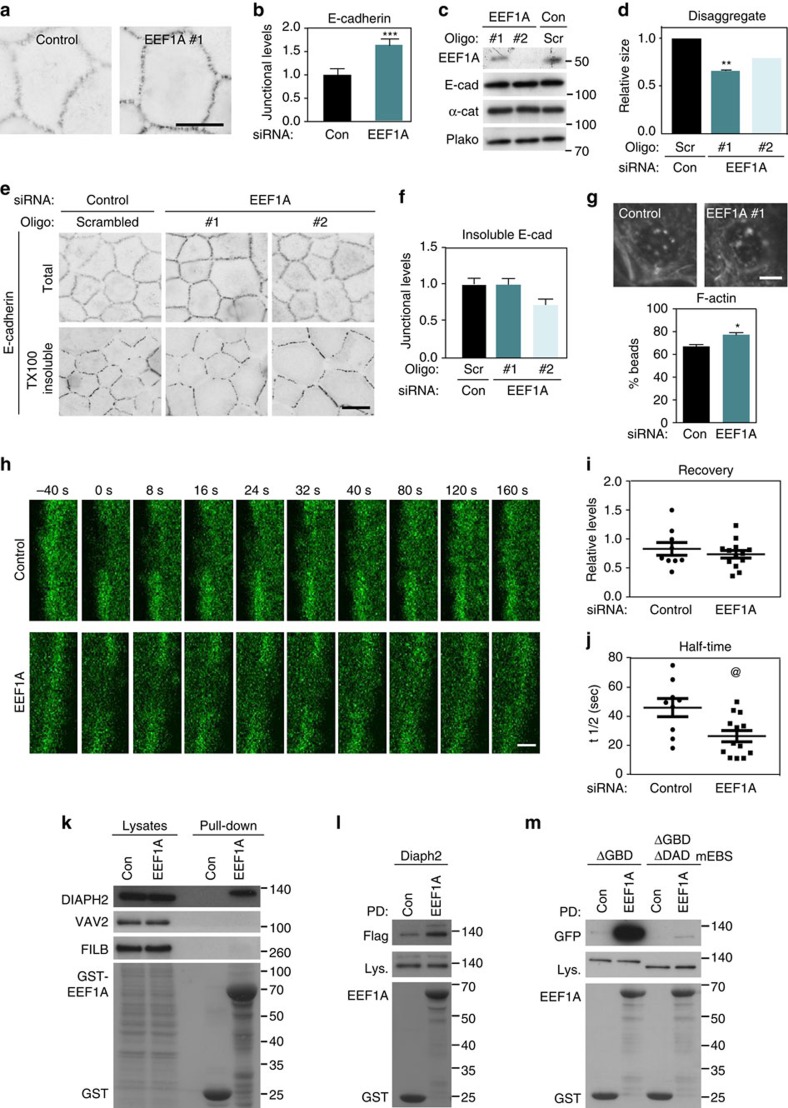
EEF1A depletion increases E-cadherin levels at junctions without strengthening cell adhesion. Keratinocytes grown in low-calcium medium were depleted of EFF1A before junctions were either induced for 30 min (**a**–**c**,**e**,**f**) or aggregation assays (**d**) and actin recruitment (**g**) performed. (**a**) E-cadherin staining images were collected and shown as representative inverted images. (**b**) E-cadherin levels at junctions were quantified as thresholded levels (% area) and normalized to controls (non-targeting oligos). (**c**) Total protein levels of cadherin and catenins following RNAi treatment. (**d**) Cells were allowed to aggregate in suspension for 2 h, followed by mechanical trituration (see Supplementary Fig. 7a). Disaggregate sizes were measured and expressed relative to controls. (**e**,**f**) Before fixation, keratinocytes were pre-extracted with TX-100-containing buffer and the insoluble pool of E-cadherin at junctions was quantified (**f**). (**g**) F-actin recruitment to clustered E-cadherin receptors around latex beads was assessed by phalloidin staining. (**h**–**j**) Dynamics of junctional actin in mature junctions was assessed. Following bleaching of selected areas at junctions, fluorescence recovery was recorded (**h**). Quantification of final fluorescence recovery levels (**i**) and the half-time required to reach the plateau in each sample (**j**) are shown. (**k**) Pull downs using GST or GST-EEF1A were performed and co-precipitated endogenous DIAPH2, VAV2 or FILB detected. (**l**,**m**) Similar experiments as in **k** were performed following expression of flag-Diaph2 wild-type (mouse mDia3, l) or activated Diaph3 (mouse mDia2, ΔGBD) and mutated at the predicted EEF1A-binding site (ΔGBDΔDAD^mEBS^; m) Molecular weight markers are shown on the right of each panel. Blots and immunofluorescence images are representative of independent replicates. Fusion proteins are detected with Amido Black staining. Scale bars, 20 μm (**a**,**e**); 10 μm (**g**); 2 μm (**h**). Error bars represent standard error of the means of independent replicates (**d** (oligo1), **f**,**g**, *N*=3; **b**,**d** (oligo2) *N*=2; **i**,**j**, control *N*=8, EEF1A *N*=13). Statistical analyses were performed with two-way Anova (**b**,**f**) or Student *t*-test (**d**,**g**,**i**,**j**). @ *P*=0.01; *P=0.007; **P=0.0002; ***P=0.002.

From this subset of cadherin regulators, we selected proteins to: (i) dissect their specific role and (ii) test the prediction that pathway finding is facilitated by the integration of phenotypic and protein network analyses. We chose to follow-up EEF1A because of its unusual phenotype, a mild increase in the levels of E-cadherin at junctions. EEF1A is an actin bundling protein also known to regulate messenger RNA (mRNA) translation at specific intracellular compartments^29^. Its function on regulation of cell–cell contacts is not known. Upon depletion of EEF1A, a small but significant increase in the levels of E-cadherin at junctions was observed (Fig. 6a,b). Following EEF1A short interfering RNA (siRNA), no changes in total protein levels of E-cadherin and associated catenins (Fig. 6c) were seen, suggesting that dysregulation of their translation is not the main determinant of the observed phenotype. The predicted outcome of higher cadherin levels at junctions as obtained with EEF1A siRNA is that cell–cell contacts are stronger. However, aggregates of cells depleted of EEF1A were more easily disrupted by mechanical stress (Fig. 6d; Supplementary Fig. 7a), indicating that junctions were not as stable as in control cells. In spite of more E-cadherin receptors at junctions, similar levels of detergent insolubility were observed in EEF1A-depleted and control cells (Fig. 6e,f). Taken together, our data strongly suggest that increased levels of E-cadherin at contact sites do not necessarily translate into more stable junctions.

To investigate this paradox closer, we evaluated the impact of reduced EEF1A levels on junctional actin. Forcibly clustering E-cadherin receptors on cells depleted of EEF1A revealed a small, but significant increase in F-actin recruitment around latex beads (Fig. 6g). We then tested whether F-actin immobilized at cadherin clusters have similar dynamic properties in EEF1A siRNA-treated and control cells. After photo-bleaching of EEF1A-depleted cells, green fluorescent protein (GFP)-actin at junctions recovered fluorescence levels similar to controls (Fig. 6h,i), but fluorescence recovery was significantly faster (Fig. 6j). Thus, following EEF1A depletion, F-actin is more efficiently recruited to cadherin receptors and yet, it does not mediate stronger adhesive contacts. Rather, the F-actin pool at junctions is more dynamic and mobile, with deleterious consequences for the stabilization of E-cadherin at adhesive sites and weaker cell–cell adhesion (Fig. 6d).

We next addressed whether signalling pathways that regulate junctions could be inferred from network and phenotypic similarity analyses. Available data sets of PPIs did not predict binding between EEF1A and other candidate proteins present in different clusters (not shown). An EEF1A-binding sequence (EBS domain) has been previously identified in Bni1p, the Rho1p effector^30^ in yeast. A similar domain is predicted in the mouse Diaph1 (aka mDia1 protein) and other DIAPH family members^30^, but binding has not yet been formally shown (Supplementary Fig. 7b). We found that EEF1A (cluster 1) co-precipitated with endogenous DIAPH2 (cluster 1), but not VAV2 or FILB (cluster 2; Fig. 6k), thereby confirming our previous results on intra-cluster protein networks (Fig. 4b).

Overexpressed wild-type mouse Diaph2 (that is, mDia3 and DIAPH2 orthologue) interacted with GST-EEF1A (Fig. 6l). GST-EEF1A also bound to diaph3 (mDia2 and DIAPH3 orthologue) in an activated status, lacking the GTPase binding domain (Δ GBD, Fig. 6m). Mutations on the EBS domain abolished this interaction (Fig. 6m). It remains to be addressed whether EEF1A interaction is also important for the function of other DIAPH family members at junctions^31^.

**Figure 7:**
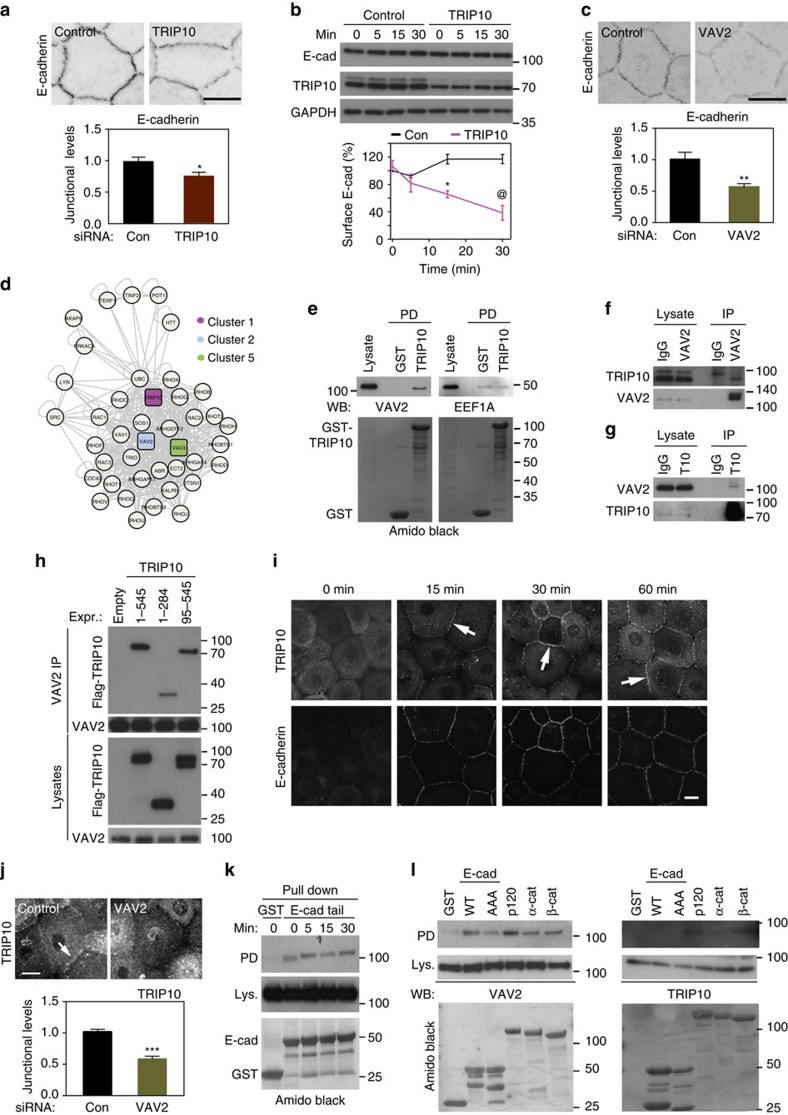
TRIP10 and VAV2 forms a functional partnership to regulate junctions. (**a**) Keratinocytes were depleted of TRIP10, junctions were induced for 30 min and samples were stained for E-cadherin. Graph shows the quantification of thresholded levels of E-cadherin at junctions (% area) normalized to controls. (**b**) E-cadherin surface levels were measured in control and TRIP10 siRNA cells during induction of cell–cell contacts. Western blots show total TRIP10, E-cadherin and GAPDH protein levels. (**c**) Cells with reduced levels of VAV2 were processed as described in **a**. (**d**) Predicted binding between TRIP10 and VAV proteins from protein–protein interaction databases. Colour code of cluster numbers is shown. (**e**) GST or GST-TRIP10 was incubated with keratinocyte lysates and co-precipitated proteins were probed for endogenous VAV2 or EEF1A. (**f**,**g**) Lysates were immunoprecipitated with anti-VAV2 (**f**) or anti-TRIP10 (**g**) antibodies and probed to detect endogenous TRIP10 or VAV2. (**h**) Cells were transfected with flag-TRIP10 wild-type and truncation mutants. Endogenous VAV2 was precipitated and flag-TRIP10 detected. (**i**) Keratinocytes were double-labelled with anti-E-cadherin and anti-TRIP10 during junction assembly. Arrows show TRIP10 localization at junctions. (**j**) Cells with reduced levels of VAV2 were induced to form junctions for 30 min and stained with anti-TRIP10 antibody. Graph shows quantification of TRIP10 levels at junctions (% area) after VAV2 RNAi. (**k**) GST-E-cadherin cytoplasmic tail was used to pull down endogenous VAV2 during a time course of junction induction. (**l**) Pull downs with GST-catenins, E-cadherin cytoplasmic tail wild-type (WT) or mutated to prevent p120 binding (AAA) were performed and probed to detect endogenous VAV2 or TRIP10. Amido black staining (**e**,**k**,**l**) denotes GST-fusion proteins. Images and blots are representative of at least three independent replicates. Statistics were two-way Anova (**a**,**c**,**j**) or Student *t*-test (**b**). *P=0.02; **P=0.0004; ***P=0.00002; @ *P*=0.004. Error bars represent s.e.m. Scale bars, 20 μm.

Other validated proteins identified in the screen are TRIP10 and VAV2. TRIP10 is a F-Bar-containing protein that participates in cadherin trafficking in Drosophila, *Caenorhabditis elegans* and mammalian tumour cell lines treated with growth factors^32^,^33^,^34^. In contrast to EEF1A, reduced levels of TRIP10 (Cluster 1) led to a small, but significant decrease of E-cadherin at newly formed junctions (Fig. 7a). This occurred without overall changes in the levels of cadherin or catenins (Supplementary Fig. 7c). Consistent with its role in DE-cadherin transport, TRIP10 appeared to modulate *de novo* cadherin recruitment to junctions (Fig. 7b). In control keratinocytes, there was a 20% increase in surface levels of E-cadherin upon induction of cell–cell contacts. TRIP10 depletion led to significantly reduced E-cadherin surface levels after 30 min of calcium switch, but no changes in total cadherin levels (Fig. 7b). Our data suggest that in the absence of TRIP10, there is no further recruitment of intracellular pools or stabilization of cadherin at junctions. E-cadherin could be internalized by different mechanisms^35^, but fails to redistribute to cell surface and junctions.

Depletion of the Rho GTPase GEF VAV2 also reduced E-cadherin levels at junctions (Fig. 7c) without interfering with cadherin and catenin levels (Supplementary Fig. 7d). An inter-cluster network between TRIP10 (cluster 1) and VAV2 (cluster 2) or VAV3 (cluster 5) was predicted in databases, but previously unknown (Fig. 7d). As both TRIP10 and VAV2 are necessary for stable cadherin adhesion during junction assembly, their predicted binding implies that these two proteins could cooperate functionally during junction assembly. GST-TRIP10 pulled down endogenous VAV2 from keratinocyte lysates, but not EEF1A (Fig. 7e). In addition, endogenous TRIP10 and VAV2 were co-immunoprecipitated using antibodies against either protein (Fig. 7f,g). VAV2 binding was mapped to the C-terminal domain of TRIP10 (Fig. 7h), which lacks the FCH (Fes and CIP4 homology) domain; disrupted F-BAR domain) but contains the HR1 (protein kinase C-related kinase homologous region 1) and SH3 domain. The interaction between TRIP10 and VAV2 is of functional significance, as during cell–cell contact assembly, TRIP10 was progressively recruited to junctions and the recruitment was dependent on VAV2 (Fig. 7i,j).

VAV2 associated with E-cadherin cytoplasmic tail in the absence of cell–cell contacts and this interaction was maintained during junction assembly (Fig. 7k). VAV2 was able to interact with p120^CTN^ strongly^36^, but also with α-catenin and β-catenin to a lesser extent (Fig. 7l). E-cadherin tail unable to interact with p120^CTN^ (E-cad AAA) co-precipitated with VAV2, suggesting that there are p120^CTN^-independent interaction of VAV2 with cadherin complexes in epithelia (Fig. 7l, Supplementary Fig. 7e; ref. 37). Endogenous TRIP10 bound to p120^CTN^ and β-catenin, but not E-cadherin tail (Fig. 7l), indicating that TRIP10 did not stably associate with cadherin tail under the experimental conditions used. Thus, VAV2 and TRIP10 interact functionally and biochemically in the stabilization of E-cadherin complexes, potentially in cooperation with p120^CTN^.

## Discussion

Expanding the repertoire of cytoskeletal proteins that support cadherin adhesion is clearly important^16^,^18^, and extensive progress has been made towards this goal^4^,^6^. Deciphering how the functions of cytoskeletal proteins are coordinated in epithelial cells is a major step forward to investigate dynamic processes such as cadherin receptor clustering and movement along the lateral domain^8^, junction elongation/shrinkage, epithelial sheet folding and tubulogenesis^4^,^5^. Here we address the challenge to infer pathways among actin-binding proteins likely to modulate cell–cell adhesion. We identify: (i) cytoskeletal proteins as new regulators of cadherin adhesion, (ii) subsets of proteins functionally linked during junction assembly and (iii) meaningful combinatorial profiles of cytoskeletal functions required for stabilization of cell–cell contacts. Our data builds on from important previous studies on DE-cadherin adhesion and junctional protein networks^16^,^18^,^38^ to highlight core regulators of cadherin cell–cell contacts.

Using a semi-automated quantification of epithelia-specific structures, candidate proteins were selected as potential junction regulators and a subset (49 proteins) further validated with individual siRNA oligos. The phenotype of the majority of the proteins depleted in the screen is a reduction in the levels of E-cadherin or F-actin at junctions. However, an unusual phenotype is also observed: an increase in the localization of E-cadherin at contact sites, which is predicted to strengthen cell–cell adhesion. Among those, a novel regulator of E-cadherin adhesion is identified as EEF1A, whose depletion enhances levels of E-cadherin receptors at junctions and F-actin recruitment. EEF1A is an actin binding and bundling protein that has a number of other cellular roles, including its canonical function to interact with amino acid-loaded transfer RNAs and their deliver to ribosomes^29^. Strikingly, with reduced EEF1A levels, higher concentration of junctional E-cadherin does not correlate with stronger adhesive function, as assessed by resistance to mechanical stress and receptor detergent insolubility. This unexpected finding challenges the notion that the strength of cell–cell adhesion correlates with the amount of receptors at junctions. The reasons for this paradox merit further studies, but it might involve: (i) interference of untethered E-cadherin with stabilized E-cadherin complexes^8^, (ii) inappropriate remodelling of actin filaments at cell–cell adhesive sites or (iii) insufficient incorporation into clusters at junctions. Indeed, junctional actin in EEF1A-depleted cells shows altered dynamics, with faster recovery following photo-bleaching compared with control keratinocytes.

Strong correlations between the actin remodelling function of a particular protein and its impact on cadherin adhesion are identified here. Robust junctional defects are observed by depletion of proteins that cap, sever or depolymerize F-actin. In addition to the known significance of actin polymerization^4^,^6^, our data highlight the importance of controlling filament length to stabilize E-cadherin contacts and the unappreciated role of other actin functions^39^. In contrast to the strong defects observed by perturbing filament length, stability and contraction, depletion of intracellular trafficking proteins leads to milder perturbation of contacts. The latter is consistent with the diversity of cellular events that control E-cadherin turnover and delivery to junctions^35^.

From the identification of unknown cadherin regulators, the challenge is to identify how regulators are integrated in discrete pathways. We surmise that the phenotypic clustering of our data set facilitates the enrichment of distinct pathways that share the same extent of disruption of E-cadherin or junctional actin. The novelty of our approach is to couple functional properties with standard phenotypic clustering analysis to guide and validate pathway enrichment. Pathway identification has been previously achieved using double-knockdown screens or by predictions that pathways segregate within a single phenotypic cluster to regulate cell morphology or GTPase regulation^19^,^28^,^40^. Consistent with the above predictions, we find a statistically significant concentration of interacting partners within all phenotypic clusters as compared with the overall protein interaction landscape. An example is a novel interaction between EEF1A and DIAPH2 (both found in cluster1). DIAPH2 (aka mDia3) is a formin family member^31^, whose function is not well characterized. A binding site for EEF1A is found in DIAPH2, consistent with the EBS domain found in the yeast homologue Bni1p^30^,^41^. Our finding that endogenous DIAPH2 interacts with EEF1A directly or indirectly may suggest a scaffolding or regulatory role of signalling by DIAPH proteins in junction regulation.

Discrete interaction networks within each cluster may be organized in different pathways with similar output (that is, level of cadherin disruption), but controlling distinct cellular processes (that is, trafficking or actin bundling). Yet, the progressive perturbation of E-cadherin adhesion found in different phenotypic clusters (that is, strong to mild perturbations) suggests an alternative distribution of interacting proteins and pathway components that regulate junctions. Indeed, a high level of interconnectivity of known junction regulators and interacting proteins is observed among different clusters: TRIP10 and WAS (Clusters 1 and 5, respectively); or VCL (Cluster 3), TLN1 (Cluster 8) and VASP (Cluster 6). These results are compelling to suggest that distinct nodes of a signalling pathway that regulate cell–cell contacts may be found in separate phenotypic clusters.

To validate the existence of pathways across different clusters, a novel functional partnership is identified that stabilizes cadherin contacts: TRIP10 and VAV2. Both proteins participate in junction disruption by distinct stimuli^32^, but their functions in cell–cell contact assembly/homeostasis have not been previously shown. TRIP10 participates in EGF-dependent E-cadherin internalization^32^ and Cip4, its Drosophila orthologue, modulates DE-cadherin endocytosis^34^. In contrast, junction assembly requires mostly transport of intracellular pools of E-cadherin to the surface, recyclling and their stabilization at junctions: TRIP10 appears to participate in these processes. VAV2 is an activator (exchange factor) of Rho GTPases^42^ and the latter have important roles in different steps of junction assembly, maturation and disruption. The partnership of VAV2 and TRIP10 identified here is functionally and clinically relevant, as both have important functions in epithelial differentiation and morphogenesis^43^,^44^,^45^,^46^, and participate in oncogenic pathways leading to cell invasion^32^,^47^,^48^,^49^,^50^.

In spite of belonging to different phenotypic clusters, TRIP10 (Cluster 1) and VAV2 (Cluster 2) interact functionally: (i) they are found as a new protein complex; (ii) depletion of either protein reduces junctional E-cadherin and (iii) VAV2 is necessary for TRIP10 localization at cell–cell contacts. These results indicate that VAV2 appears to be upstream of TRIP10 in junction regulation. VAV2 has been shown to interact specifically with the cytoplasmic pools of p120^CTN^ and β-catenin^36^,^51^,^52^. In contrast, in keratinocytes VAV2 interacts with E-cadherin tail at steady state and during junction assembly. There is a large pool of VAV2 associated with p120^CTN^ and a smaller pool that interacts with E-cadherin complexes (even in the absence of p120^CTN^ binding), α-catenin and β-catenin. Mechanistically, VAV2 stabilization of newly formed junctions may require a transient positioning of TRIP10 at cell–cell contacts to mediate E-cadherin trafficking and/or to remodel actin filaments. Indeed, VAV2 associates with TRIP10 C terminus, which includes the binding region of the small GTPase Cdc42 (refs 53, 54). Thus, the VAV2-dependent localization of TRIP10 at new junctions may coordinate TRIP10 binding to GTPases activated locally. The novel interplay between GTPase signalling and TRIP10 (via the GEF VAV2) or EEF1A (via the GTPase effector DIAPH2) highlight the power of our analyses to infer pathways. Future studies will define further mechanisms of how TRIP10 and EEF1A functions are coordinated with GTPase-dependent pathways to modulate cadherin adhesion.

Our data demonstrate that coordination of both known and unknown junction regulators can be derived by functional annotation, interaction profiling and phenotypic similarity analysis. The framework identified here provides an exciting platform to identify new molecules that modulate junctions and to integrate small GTPase signalling with trafficking and actin filament remodelling. Our study presents a powerful strategy to dissect hierarchical relationships that control cadherin stabilization with significant implication for research in epithelial morphogenesis, homeostasis and diseases.

## Methods

### Cell culture

Normal human keratinocytes from neonatal foreskin (strain Sf, passages 3 to 5) were cultured on a mitomycin C (Sigma)-treated monolayer of 3T3 fibroblasts at 37 °C and 5% CO_2_ in standard medium as described^55^. Unless otherwise stated, keratinocytes were seeded in standard calcium medium (containing 1.8 mM CaCl_2_), transferred to low calcium medium (0.1 mM CaCl_2_ and fetal calf serum depleted from divalent cations) and grown until confluence^56^. For siRNA screen keratinocytes were seeded at 2.2 × 10^3^ per well on 96-well plates as above and grown in low calcium medium and, when a confluent monolayer is formed, treated with different siRNA oligonucleotides. Induction of cell–cell contacts (calcium switch) was done for 30 min by addition of 1.8 mM CaCl_2_ or by transferring cells to standard calcium medium (RNAi screen).

Treatment with different drugs was performed in confluent cultures in low calcium medium by pre-incubation for 30 min with PMA (4β,9α,12β,13α,20-pentahydroxytiglia-1,6-dien-3-one 12-tetradecanoate 13-acetate, Sigma), 5 μM Y27632 (Sigma) or for 60 min with 50 μM blebbistatin (Merck). Junctions were induced for 30 min in the presence of the drugs.

### Transfections and constructs used

Mammalian expression vectors were transfected using TransIT (Mirus Bio LCC), JetPRIME (Polyplus transfection) or Viromer reagent (Lipocalyx). constructs were expressed overnight.

Constructs used were: activated Arf6 (pCS2-HA-Arf6^Q67L^) (ref. 57), pCDNA3.1.GFP-β-actin (gift from M. Bailly, University College London), pGEX2T-TRIP10, pRK5-Flag-TRIP10 1–545, pRK5-Flag-TRIP10 1–284, pRK5-Flag-TRIP10 95–545 (ref. 58) (all gifts from P.Aspenstrom, Karolinska Institute) and pGEX 6P-E-cadherin tail (mouse)^59^. For Dia constructs the current nomenclature (http://www.ncbi.nlm.nih.gov/gene) was used throughout. Full length mouse Diaph2 (pCMV-3Xflag-1A-mDia3) is based on a synthetic construct (accession number BC160217). Mouse Diaph3 (mDia2, accession number AF094519) was truncated at the N terminus (aa 1–254) to partially delete the GTPase-binding domain (GBD; pEF_m_-EGFP-mDia2 ΔGBD), and at the C terminus (from aa 1041) to delete the DRF auto-inhibitory domain (DAD; pEF_m_-EGFP-mDia2 ΔGBDΔDAD)^60^. In addition, Diaph3 was mutated on the predicted EBS to introduce glycines (Y713G, E714G, K715G and R717G) and generate ΔGBDΔDAD^mEBS^ that is unable to interact with EEF1A.

Mouse EEF1A was subcloned into pGEX2T between BamHI and EcoRI restriction sites using PCR. Point mutations to E-cadherin cytoplasmic tail were made to obtain triple alanine mutations starting at amino acid 764 (E-cad AAA) to prevent binding to p120^CTN^ (ref. 37). GST-fusion proteins were produced using standard protocols and stored in aliquots at −80 °C.

### Immunofluorescence and microscopy

Antibodies used for fluorescence and western blots were against E-cadherin (HECD-1; 1:1,000–1:3,000), tubulin (Tub2.1, Sigma; 1:1,000), VAV2 (EP1067Y, Abcam; 1:2,000–1:20,000), TRIP10 (F-10, St Cruz; 1:200–1:10,000). Catenin p120^CTN^ (98/pp120, BD Biosciences; 1:500), α-catenin (1:1,000) and plakoglobin (1:1,000; ref. 61), EEF1A (D10A5, Cell Signalling, 1:100–1:200), DIAPH2 (NBP1-85217 Novus Biologicals and ab102841, Abcam, 1:250), FILB (A301-726A, Bethyl Laboratories, 1:10,000) and GAPDH (6C5, Abcam; 1:100,000). Secondary antibodies were bought from Jackson ImmunoResearch. F-actin was labelled using AlexaFluor 488 phalloidin (Invitrogen; 1:1,000–1:3,000) or Phalloidin–Atto 565 (Sigma; 1:1,000). DNA was labelled using DAPI (Sigma, 1:3,000). Uncropped western blots can be found in Supplementary Figs 8 and 9.

Unless otherwise stated, keratinocytes were fixed in 3% paraformaldehyde for 10 min. Cells were permeabilized with 0.1% Triton and blocked with 10% FCS for 10 min and stained as described^56^. Non-screen images were acquired on a Zeiss inverted 510 LSM laser-scanning confocal (Carl Zeiss) using a 63 × /1.4 Plan Apochromat objective or with an Olympus Provis AX70 microscope coupled to a SPOT RT monochrome camera using Simple PCI software (Hamamatsu, Japan). Aggregation assays were imaged using a phase contrast Olympus CKX41 microscope and a Colour View IIIu camera linked to Sort Imaging System software. Fluorescence recovery was performed on an inverted confocal microscope (LSM-510; Carl Zeiss) using ZEN software.

### Image processing

In-house segmentation tools were developed (Supplementary Fig. 1) to generate three experimental parameters representative of E-cadherin (E-cad), junctional actin (that is, co-localizing with E-cadherin and named Jun-A) or cytoplasmic actin (that is, excluding junction and nucleus area; Cyt-A). Analysis was designed using MBF-ImageJ (http://imagej.nih.gov/ij/) and Metamorph 6.2 (Molecular Devices). E-cadherin image was thresholded to minimize contribution of cytoplasmic staining (E-cad parameter); positive value for the E-cadherin parameter may reflect increased cadherin levels at junctions and/or cytoplasm. Thresholded E-cadherin image was used as a mask to segment junctional actin pool (Jun-A) from the corresponding image stained with phalloidin (total F-actin). Cytoplasmic actin (Cyt-A) was obtained by subtraction of E-cadherin mask and nucleus image from the total F-actin image. As a confluent monolayer was used in all experiments, no significant change in cell shape in two-dimensional space is observed (that is, see ref. 62), that would affect E-cadherin mask and thus E-cad and Jun-A parameters. These parameters assess effectiveness of junction assembly by an intensity-based approach; their caveat is the inability to identify changes in the shape, length or continuity of E-cadherin staining.

To validate the new segmentation tool and ensure the dynamic range of the measurements, images were obtained with different levels of disruption of the actin cytoskeleton or E-cadherin at junctions and different readouts assessed. Algorithms were validated to demonstrate no correlation with cell number. Optimized algorithms were incorporated into a custom-made automated program to analyse images obtained in the screen.

To quantify the amount of specific proteins at junctions, E-cadherin images were individually thresholded to minimize contribution of cytoplasmic staining and maximize junction coverage. The thresholded binary E-cadherin image was dilated and used as a mask to segment the junctional TRIP10 pool from the corresponding TRIP10 image. A global threshold across all experimental conditions was applied to the segmented TRIP10 images to measure the TRIP10 junction coverage and calculate % thresholded area. Graphs were made using Excel or GraphPrism and figures were assembled using Illustrator and Photoshop.

### RNAi screen

The custom-made Actinome library targeting 327 known or predicted actin-binding proteins^10^ (Thermo Scientific Dharmacon) was made up of SMARTpools (pool of four different siRNA oligos targeting each mRNA) and an accompanying validation library (four single oligos per mRNA). The primary screen was carried out as three independent replicates: keratinocytes were transfected with 100 nM of SMARTpool siRNA using RNAifect (Qiagen). As controls, a pool of scrambled oligos (siCONTROL siRNA, a RISC-free siRNA) was used. siTOX and INCENP (Dharmacon) were used as positive markers for transfection, and each plate was visually checked for cell death (siTOX) or increase in cell size (INCENP) before analysis. In addition to these transfection controls on each plate, there were multiple wells as negative controls (cells untransfected and not induced to assemble junctions) and positive controls (cells untransfected with junctions induced). After 72 h incubation, cell–cell contacts were induced with fresh standard calcium medium (calcium switch) for 30 min before fixation. Our previous studies show that junctions assembled for 30 min in keratinocytes are sufficient to form polarized cuboidal cells and reorganize the actin cytoskeleton as observed in other simple epithelial cells^62^,^63^. Plates were prepared for immunofluorescence using the standard protocol (above) and mounted in 50 μl Mowiol and 1% 1,4-diazabicyclo[2.2.2]octane.

Screen images were acquired on a GE Healthcare InCell Analyser 3000 line scanning confocal system using a 40 × /0.8 objective and converted to 16 bit TIFF format using InCell exporter and images were renamed for documentation and batch processing using Irfanview (http://www.irfanview.com). Quality control was carried out visually to eliminate any image in which cells were not 90% confluent or in which there were problems with acquisition (that is, bright/dead cells that would skew the quantification).

### RNAi screen analysis

The semi-automated system used selected controls, segments the different experimental parameters from the raw images. The data set obtained was tested for Gaussian and log-Gaussian distribution (see below). *Z*-scores in each experiment were calculated and normalized to controls in each plate^24^ due to the high likelihood of obtaining phenotypes with a targeted library and the non-parametric distribution of the dataset. Final *Z*-score was computed as the median of the three independent experiments performed during the primary or validation screens. A cutoff was decided based on standard deviation (+/−1.65 STD) to select samples with E-cad, Jun-A or Cyt-A *Z*-scores above or below the cutoff as candidate proteins. A total of 156 candidate proteins were selected for further validation using four independent siRNA oligos, and from this subset, 49 proteins were further investigated.

Validation screen was performed in triplicate and processed essentially as described for the primary screen. However, the same cutoff could not be used in the validation screen because the amplitude of the responses (relative to positive and negative control values) was lower. This may reflect the biological variability of primary cells and the fact that single oligos were used rather than a pool of four oligos in the primary screen (predicted to deplete proteins more efficiently). Samples with two or more *Z*-scores outside cutoff in the same direction (either positive or negative) were considered validated. A few samples with ambiguous status (two positive *Z*-scores and two negative *Z*-scores) were selected for additional experiments to confirm their role. We were interested in defining junction regulators with a milder rather than very strong phenotype (that is, very high or very low *Z*-scores), as this may suggest a non-specific disruption of the actin cytoskeleton by depletion of a core actin regulator. Milder phenotypes are a bone fide representation of the additive and cooperative actions of proteins towards junction stabilization. Further characterization of hits certified that levels of proteins found in cadherin complexes and Rho GTPases were not altered upon depletion of proteins analysed in more detail.

### Functional analysis

For the sake of consistency, the notation of gene names is kept throughout the text and figures. The three approaches were taken to evaluate functional enrichment of specific functions in the 156 candidate proteins. First, GO terms were obtained and the frequency of a specific attribute plotted. However, there was no significant enrichment of annotated functions using GO classification (data not shown). Second, specific actin remodelling functions were manually curated from databases and literature so that each candidate protein was ascribed one or more actin functions that they regulate. All reported activities for a given protein were computed in the 13 functional classes by manual curation of the literature. Graphs were prepared to show the distribution of 13 distinct actin functions within a cluster in two ways: as a percentage of functions allocated to the cluster or as a percentage of all proteins with a particular function. Enrichment was considered when representation of a class of actin function in a cluster was ≥25% of total number of proteins classified in class.

Finally, publicly available PPI data for the candidate proteins data set were downloaded from IntAct, MINT, BIOGRID, DIP, HPRD and Reactome (level 1 and 2 interactions; total of 68,549 interactions). Each protein was assigned to an Ensembl62 gene using Ensembl62's external references from Biomart. PPI maps using the data sets obtained were produced using Cytoscape. Global interaction network was obtained for all candidate proteins and within each cluster. To facilitate pathway finding, we focused on addressing sub-networks that may regulate a particular actin remodelling property enriched in specific clusters.

### Phenotypic clustering

As the data set do not follow a Gaussian distribution (see Statistic section), we optimized the methodology to classify the candidate proteins into distinct groups with functional relevance for the regulation of E-cadherin and F-actin at junctions. Each of 156 data points in the candidate protein data set is a three-dimensional vector that contains the *Z*-scores for E-cad, Jun-A and Cyt-A. Using MATLAB (The MathWorks), we evaluated three different methods^26^,^64^ (K-means, hierarchical or spectral clustering) and two different distances (Euclidean and Mahalonobis). The Mahalanobis distance^26^ between any two data points *X* and *Y* is computed here by *d*_*M*_*(X,Y)*, which is transformed into a similarity measure via *s(X, Y)=*exp*(−(d*_*M*_*(X, Y))*^2^*/*(2*σ*^2^)), where we set *σ*=1. For spectral clustering, we used the mutual k-nearest neighbour graph with 30 neighbours and computed the normalized graph Laplacian. For optimization of the parameters, we used a modified version of MatLab GUI (Graph Demo), which can be assessed in http://www.ml.uni-saarland.de/code/GraphDemo/GraphDemo.htm.

We tested permutations of the best method (Hierarchical or Spectral), distance (Euclidian or Mahalanobis) and number of clusters using the silhouette plot method^25^, which provides a graphical representation to assess clustering validity. Each data point is given a silhouette value that ranges from −1 to +1. A positive value provides evidence that a point is correctly classified in its cluster, while a negative value indicates that the point might be better assigned to a different cluster. Initially, *k*=2, ...., 12 clusters were evaluated selecting the plot with least misclassifications.

To further refine the best clustering output, we assumed that biological significance would be translated as enrichment of phenotypes and protein functions in specific clusters (Supplementary Fig. 6). Spectral clusters obtained using Euclidian or Mahalanobis distances were re-tested the pattern of segregation of the different parameters (E-cad, Jun-A or Cyt-A) and the enrichment of specific functions of actin remodelling. This analysis confirmed that the combination of Spectral with Mahalanobis was the best methodology tested. Finally, the appropriate number of clusters was re-evaluated using Silhouette plots (Supplementary Fig. 6e; Fig. 3) to obtain the final data set. Very few data points had a negative Silhouette value, indicating that samples were correctly classified in their respective clusters.

Finally, to validate the optimal clustering obtained, two tests were performed: (i) functional enrichment (see above) and (ii) a similarity score was obtained based on the topology and protein interaction network of the candidate protein data set (Supplementary Table 1). The assumption for the latter is that any functional similarity above random must arise from more functionally similar genes being placed in the same cluster as compared with ‘random'. The main strength of this approach is that the analyses only included genes from the screen and with functional annotation in the Kernels. Thus, functional similarity above random will not be due to inappropriate choice of a background set of genes.

Briefly, around 10,000 randomized clusters were formed by partitioning the candidate proteins found in the RNAi screen into clusters of the same size but with random, distinct composition of proteins. This was done in order to preserve the number and size of the clusters to be the same as the optimized experimental clusters obtained above. The network of PPIs of the candidate proteins was converted into a Kernel using a Commuter time Kernel (CK)^27^,^65^,^66^ to measure the homogeneity of the clusters. For a given randomized cluster, the Commuter time (CK) score was calculated as following: for each protein in each cluster, the highest kernel similarity score to any other protein in the same cluster is obtained. CK scores for all proteins in the cluster and for all clusters were summed to get the global score (CK-max score). The density estimate of the CK-max scores was then plotted using the density function in R for all the randomly generated clusters.

### Statistical analysis

Graphs were created using Statistica 9, Microsoft Excell or MATLAB (The MathWorks). Statistical analysis was performed using SPSS Statistica (Wolfram Research Institute), PRISM or Excel. Inclusion or exclusion of data points for the different correlations is described in the respective figure legends. Test of normality used was Shapiro-Wilk W, in which the closer the value is to 1, less likely a normal distribution is (Supplementary Fig. 3). Log-Gaussian distribution was also tested, as this distribution is frequently found in biological systems: raw data values were log-transformed and corrected for skewness before applying the Shapiro-Wilk W test. The values obtained for the latter analyses (*W*<0.967 and *P*<3.4 × 10^−11^) indicate that the data do not follow a log-Gaussian distribution. Correlation analysis was done using Pearson correlation tests, followed by two-sided *F*-test to define significance (*P* value). For quantification of VAV2 and TRIP10 phenotypes, two-way Anova was done to test for significant changes between experiments and experimental conditions, followed by MatLab function ‘anovan' to obtain *P* values. The samples meet the assumptions of the test (normal distribution, equal variance, independence) and variance is not significantly different between different groups.

### Data availability

The authors declare that all data supporting the findings of this study are available within the article and its Supplementary Information files or from the corresponding author upon reasonable request. Access to the software for large-scale image analysis developed here can be obtained in the link http://www.imperial.ac.uk/bioinformatics-data-science-group/resources/software/rnaiscreener/

## Additional information

**How to cite this article:** Erasmus, J. C. *et al*. Defining functional interactions during biogenesis of epithelial junctions. *Nat. Commun.* **7,** 13542 doi: 10.1038/ncomms13542 (2016).

**Publisher's note**: Springer Nature remains neutral with regard to jurisdictional claims in published maps and institutional affiliations.

## Supplementary Material

Supplementary Information Supplementary Figures 1-9, Supplementary Tables 1-3, Supplementary Methods and Supplementary References.
