# Corrigendum: Defining functional interactions during biogenesis of epithelial junctions

**DOI:** 10.1038/ncomms14195

**Published:** 2017-01-05

**Authors:** J. C. Erasmus, S. Bruche, L. Pizarro, N. Maimari, T. Poggioli, C. Tomlinson, J. Lees, I. Zalivina, A. Wheeler, A. Alberts, A. Russo, V. M. M. Braga

Nature Communications 7: Article number: 13542; DOI: 10.1038/ncomms13542 (2016); Published: 12 06 2016; Updated: 01 05 2017

The original version of this Article contained an error in the spelling of the author Tommaso Poggioli, which was incorrectly given as Tommaso Pogglioli. This has now been corrected in both the PDF and HTML versions of the Article.

