# Supplementary material for: Defining functional interactions during biogenesis of epithelial junctions
Source: Nat Commun. 2016 Dec 6;7:13542. doi: 10.1038/ncomms13542 (PMC5150262; doi:10.1038/ncomms13542)
Supplement: Supplementary Information — Supplementary Figures 1-9, Supplementary Tables 1-3, Supplementary Methods and Supplementary References. [file ncomms13542-s1.pdf]

Supplementary figures:

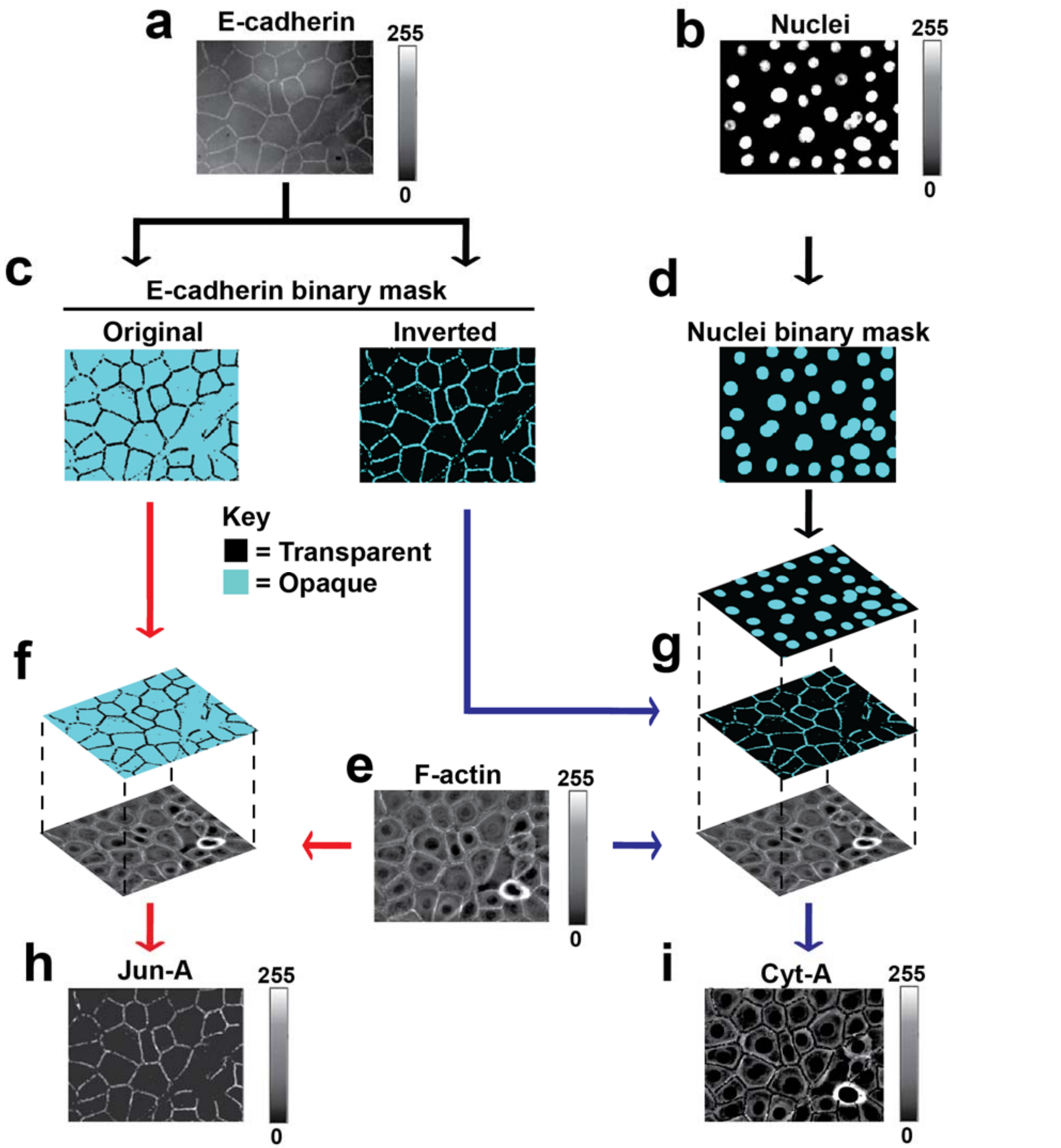

Supplementary Figure 1: Segmentation of parameters E-cad, Jun-A and Cyt-A. Keratinocytes were stained for E-cadherin receptors (**a**), nuclei (**b**) or F-actin (**e**) and processed as follows. **a**, E-cad parameter was obtained by thresholding images of cells stained for E-cadherin to minimize contribution of staining in the cytoplasm. **c-d**, E-cadherin (**a**) and nuclei (**b**) images were converted into a binary mask (black arrows; c, d, respectively). **f**, red arrows, The parameter Jun-A (**h**) was segmented to represent the F-actin pool that co-localises with E-cadherin receptors at junctions (junctional actin): the original E-cad binary mask (c) was overlaid onto the F-actin image (e) and the pixels at the intersection between the two images show F-actin at junctions (h). **g**, blue arrows, The parameter Cyt-A (**i**) represents the F-actin pool found in the cytoplasm (i.e. excludes junctional actin and any F-actin found at the nucleus). The inverted E-cad (c) and nuclei (d) binary masks were subtracted from the F-actin image (e) and the remaining pixels were defined as Cyt-A parameter (i).

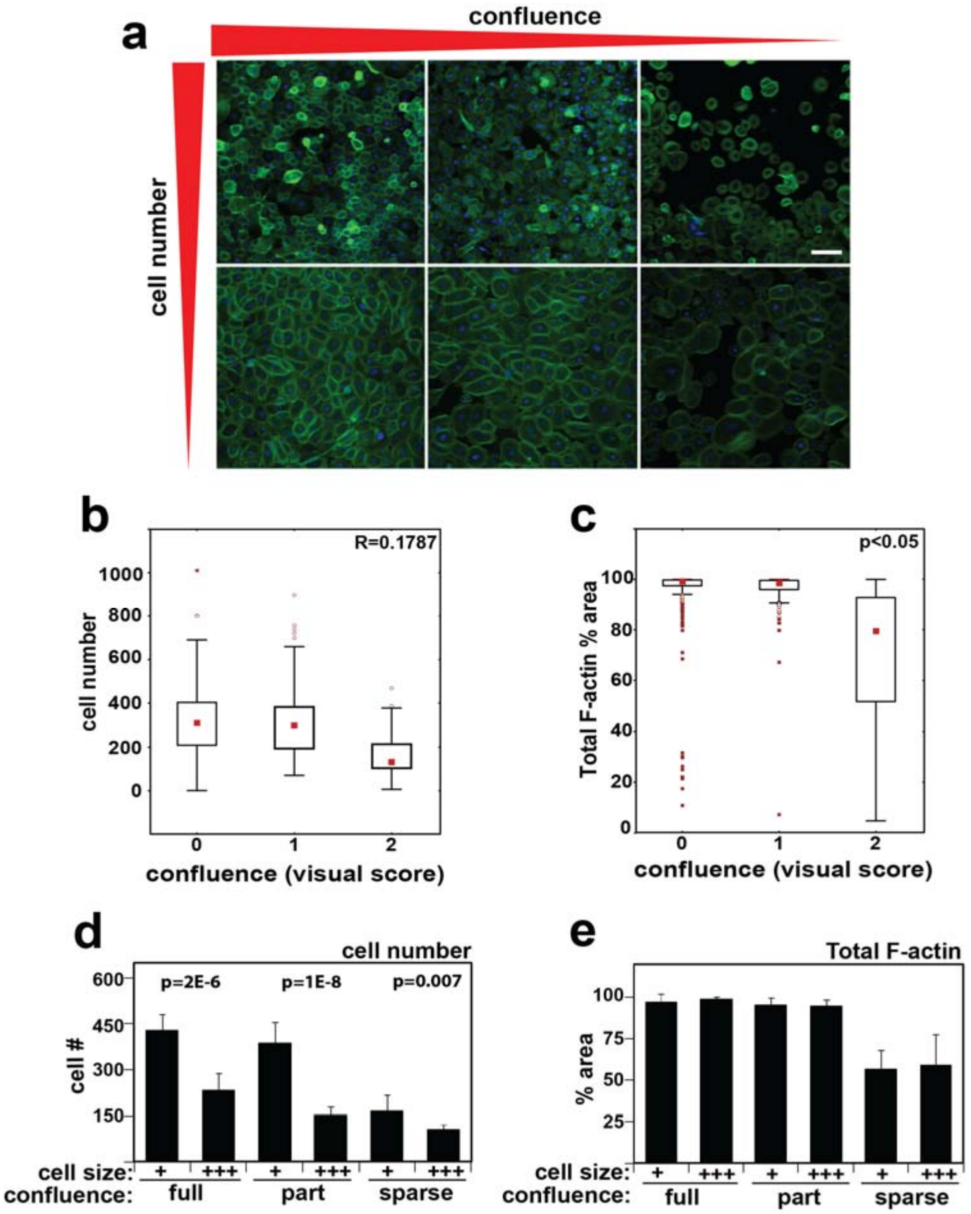

Supplementary Figure 2: Automated image quality control - determination of confluence. Gaps in the monolayer delay junction assembly and may lead to false-negative results. An automated quality control was designed to eliminate images with unsuitable confluence (i.e. containing gaps). **a,** Keratinocytes were stained for nucleus (blue) and F-actin (green). Images were selected visually to contain varying cell sizes and at different confluence. **b**-**c**, Images were visually classified as confluent (0), >90% confluent (1) and sub-confluent (2). Images classified as "2" would not be considered in the RNAi analysis. Confluence status was plotted against number of cells (nuclei counting using ImageJ, b) or the percentage of thresholded area of F-actin staining (i.e. % pixels in the image after thresholding, Total F-actin % area, c). Data shown are number of cells or total F-actin in each well, values of all three replicates were computed together (n= 981 data points). Box plots represent 25%-75% interval, dots show medium values and open circles show outliers. **d**-**e**, Images of cells of different sizes (+ to +++ representing small to large sizes) and confluence (full, part, sparse) were processed to determine number of cells by DAPI (cell #, d) or the number of F-actin pixels as percentage of thresholded area (e, Total F-actin, % area). Error bars represent standard deviation from 3 independent experiments. N= 54 images were (9 images per condition of confluence and size). As a confluent image could be observed with a reduced number of larger cells (a), the use of cell number is not ideal for confluence quality control. Instead, the % thresholded Total F-actin compared favourably with the visual score of each image (e). Scale bar=50µm.

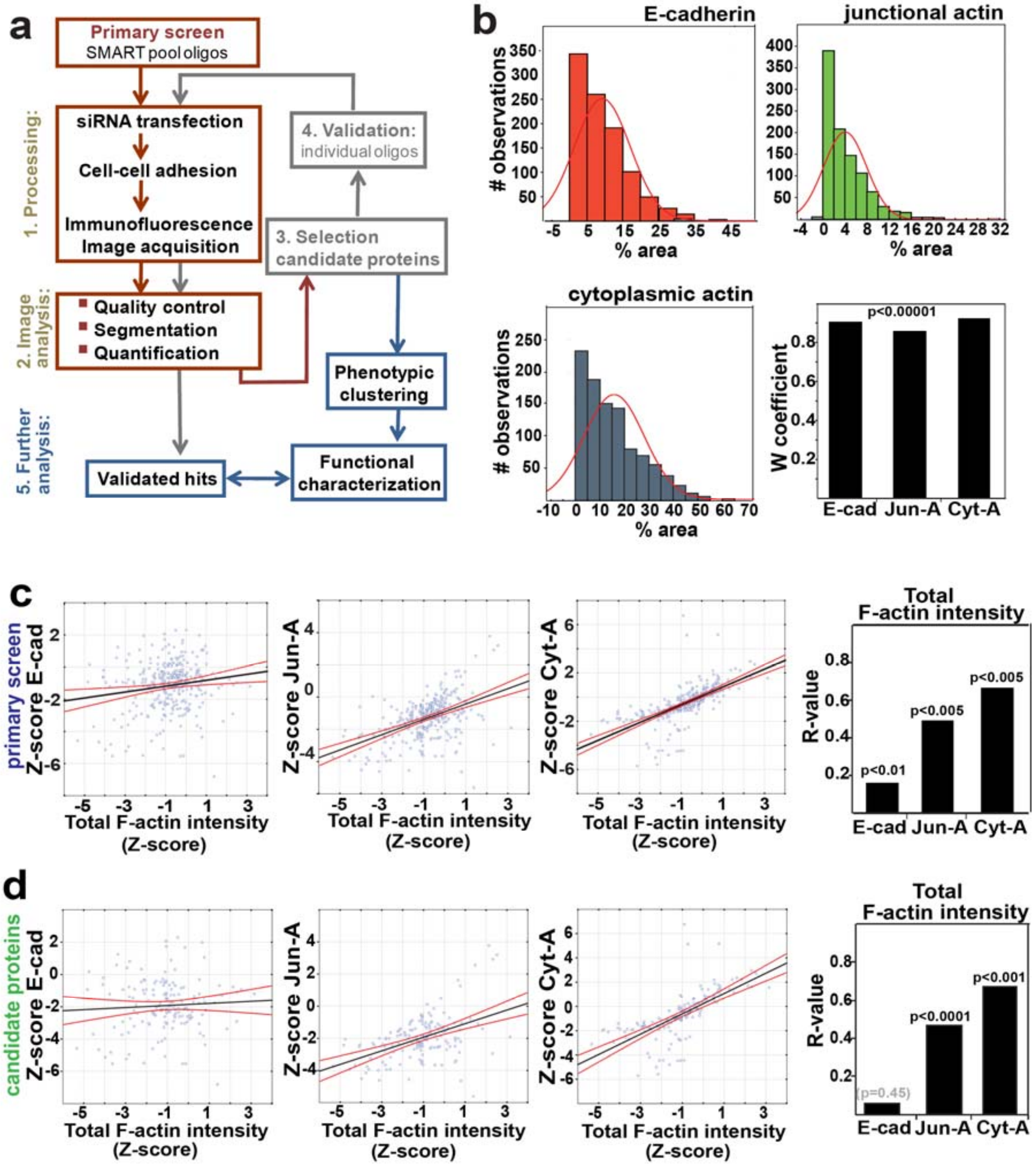

Supplementary Figure 3: RNAi workflow and correlation among different parameters. a, RNAi screen was structured in two tiers performed in triplicate experiments: primary screen (pool of four siRNA oligos against each mRNA; red boxes and arrows) and validation screen (four individual oligos tested separately for each mRNA; grey boxes and arrows). Cells were processed (step 1) and acquired images quantified as optimized (step 2; Fig.1, S1). Ranking of the median Z-scores for each parameter allowed the selection of 156 candidate proteins (step 3) to be re-tested in the validation screen (step 4), when its phenotype was reproducible with two or more oligos. Samples were analysed globally with phenotypic clustering and functional characterization (step 5; blue boxes and arrows). **b**, Non-Gaussian distribution of the parameters E-cad, Jun-A or Cyt-A obtained in the primary screen (n=981, all replicates). Shapiro-Wilkins W test was performed and W coefficient values are shown. The closer to 1 the value is, the less likely a normal distribution. **c**-**d**, Z-scores obtained in the primary (c, N=327) or candidate (d, N=156) screen datasets were plotted against the Z-scores of total F-actin intensity. Median Z-scores values of each siRNA oligo from three replicates were pooled together. Black lines represent the linear regression models and curved red lines show their 95% confidence interval. Pearson correlation coefficients (R-values) for each plot are also shown.

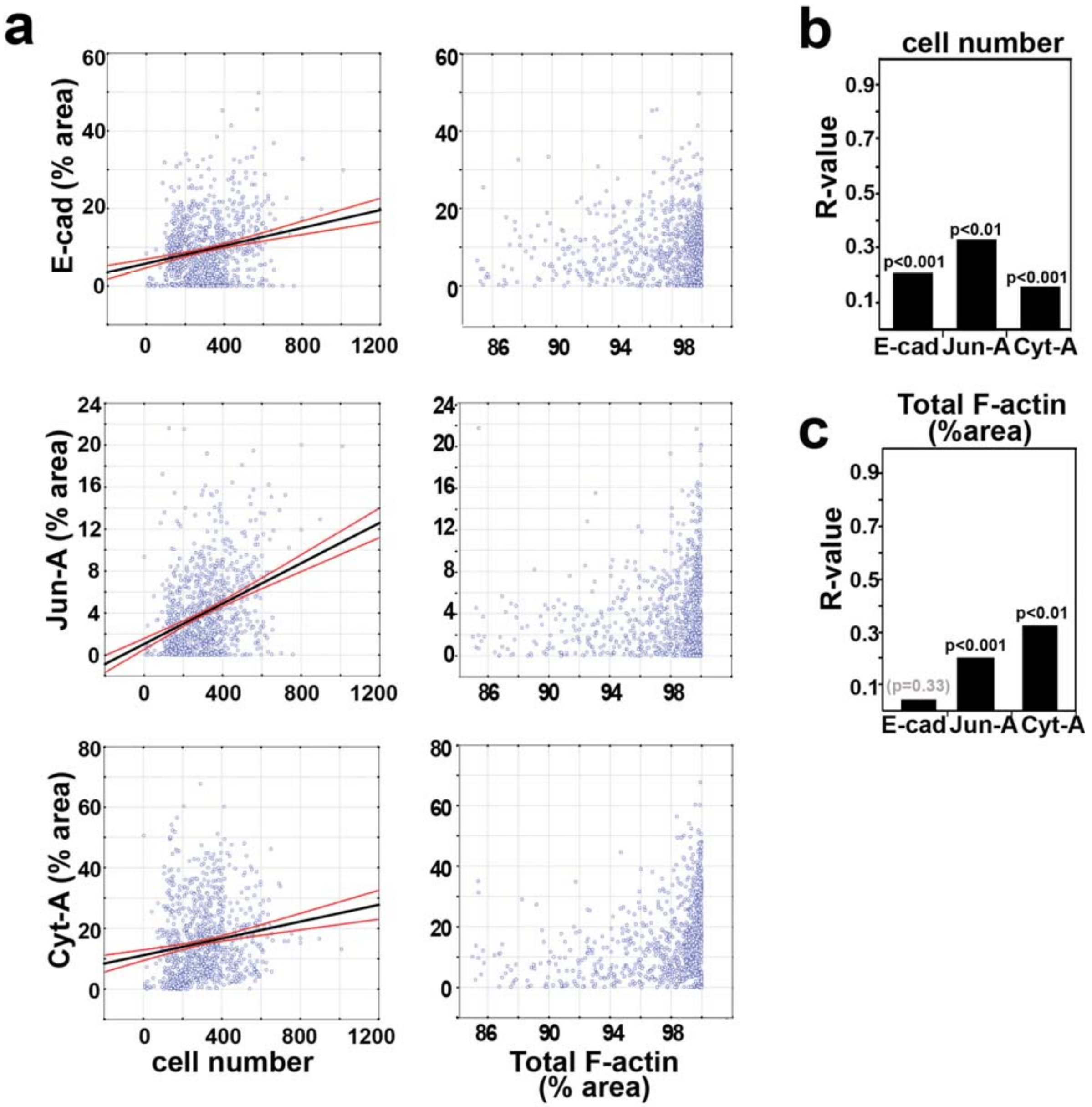

Supplementary Figure 4: Experimental parameters do not correlate with cell number or confluence. **a**, For each well of the three replicates of the primary screen, raw values of experimental parameters (E-cad, Jun-A and Cyt-A) were plotted against the number of cells or the percentage thresholded area of Total F-actin (% area). Black lines represent the linear regression models and curved red lines show their 95% confidence interval. N= 981 data points. **b-c**, Pearson correlation coefficients (R-values) between the graphs in a-b are shown.

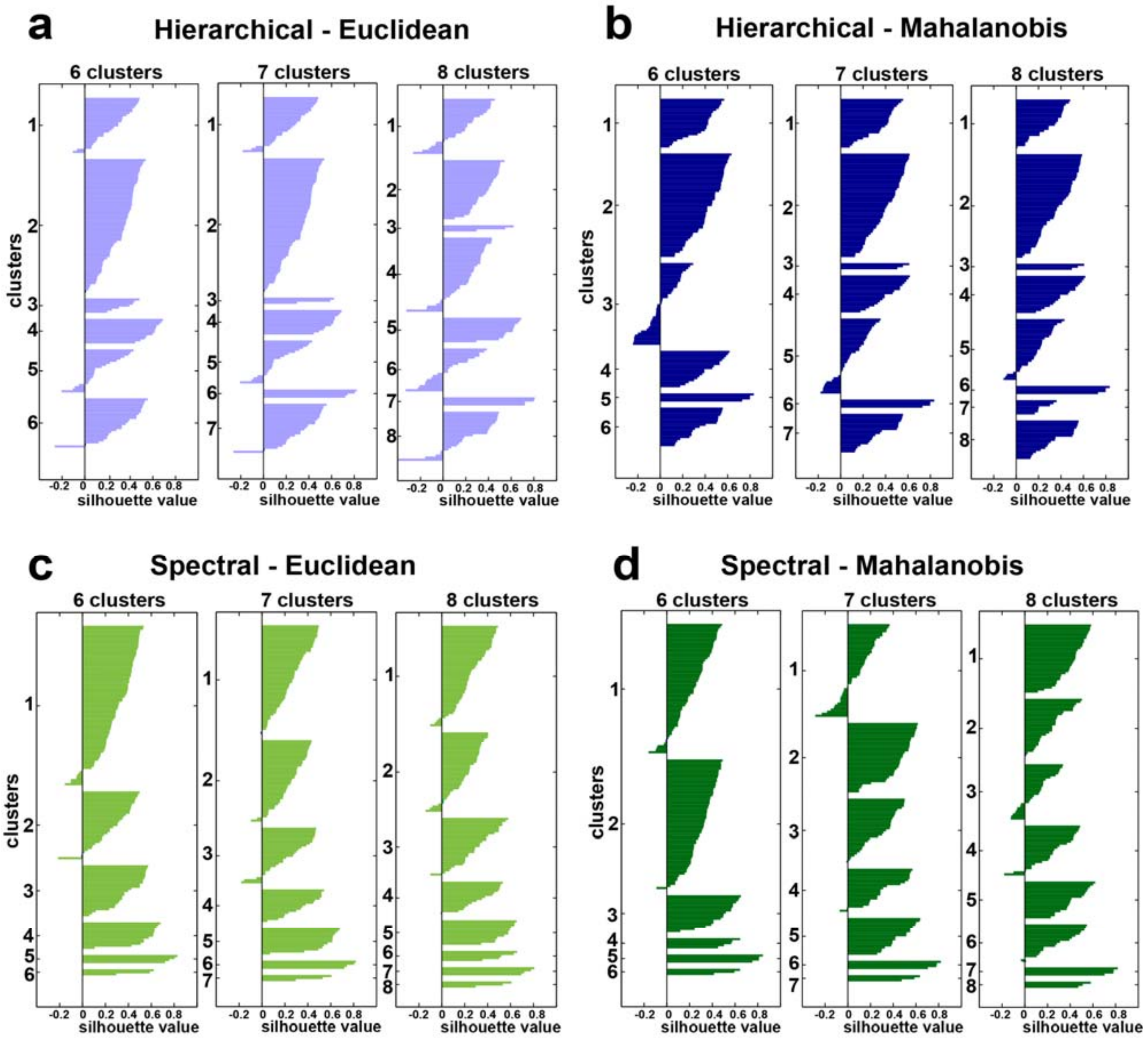

e **Spectral - Euclidean**

Number clusters	negative silhouette values		average all values
	number	average	
6	10	-0.075	0.343
7	7	-0.077	0.303
8	10	-0.070	0.309

f **Spectral - Mahalanobis**

Number clusters	negative silhouette values		average all values
	number	average	
6	8	-0.071	0.317
7	18	-0.090	0.308
8	13	-0.075	0.304

Supplementary Figure 5: Optimization of phenotypic clustering. Clustering methodology was optimized as our dataset does not follow Gaussian distribution, and thus Euclidian distance is not recommended. Candidate protein dataset was clustered using two methodologies hierarchical (a-b) and spectral (c-d) using two distances, Euclidean (a,c) or Mahalonobis (b,d). Different numbers of clusters were tested (6–8 clusters) and validated for their suitability using different approaches. **a-d**, Comparison of silhouette plots for clusters obtained with hierarchical (a,b) or spectral (c,d) methodologies. Silhouette plot shows a silhouette value (-1 to +1) for each point in the dataset measuring how similar it is to points within its own cluster compared to points in other clusters. Positive values show that the point is correctly classified in its cluster. **e-f**, Comparison of Silhouette values obtained with 6-8 clusters following spectral method and Euclidean (e) or Mahalanobis (f). Profiles obtained with spectral using Euclidean or Mahalanobis distances were further evaluated for biological significance of the clusters obtained (see Figure S6).

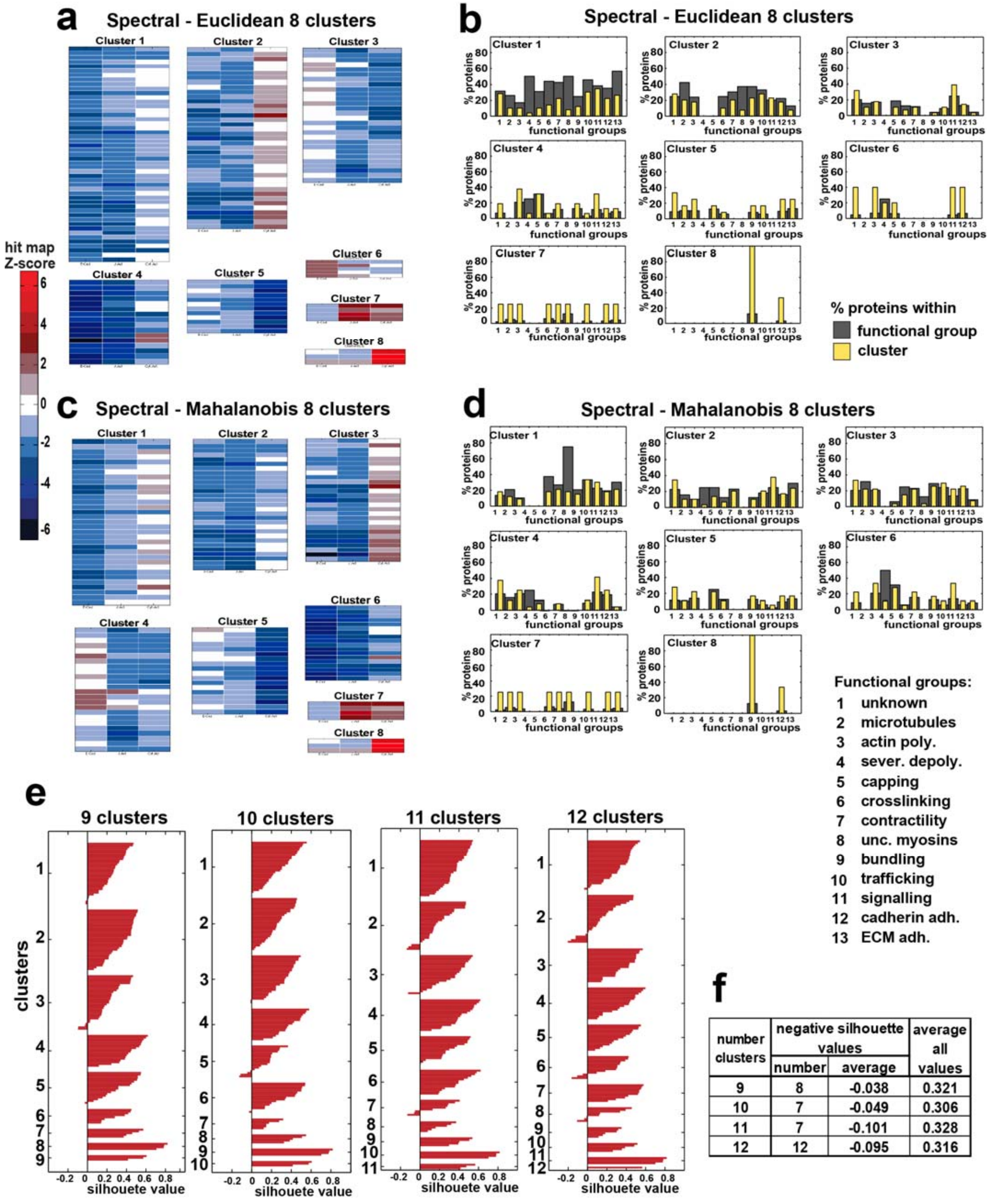

number clusters	negative silhouette values		average all values
	number	average	
9	8	-0.038	0.321
10	7	-0.049	0.306
11	7	-0.101	0.328
12	12	-0.095	0.316

Supplementary Figure 6: Suitability of phenotypic clustering methodology to enrich functional attributes of each protein. Candidate protein dataset was clustered using Spectral and the distances Euclidean (a-b) or Mahalanobis (c-d). **a**, **c**, Heat maps of the Z-scores of E-cad, Jun-A and Cyt-A parameters are shown for 8 clusters. Mahalanobis distance yielded better segregation of distinct phenotypes in specific clusters. **b**, **d**, To demonstrate that the phenotype segregation reflected enrichment of specific actin functions, manual curation of the literature was performed and each of the candidate protein was attributed one or more functions on actin remodelling (13 distinct functional groups; X-axis). Enrichment of specific functions is shown in two ways. First, the percentage of an actin function found in a cluster compared to all proteins within each functional group (grey bars, i.e. all proteins known to bundle filaments). Second, data represent the distribution of an actin function among all functional groups found within a given cluster (yellow bars). Mahalanobis distance provided the best enrichment of phenotypes and actin functions, particularly for the larger clusters (1-6). **e**-**f**, Further optimization was performed to test numbers of clusters (9-12 clusters, n=156 data points). Silhouette graphs (e) and comparison of values (f) are shown. Further analyses and optimization was done using 9 clusters.

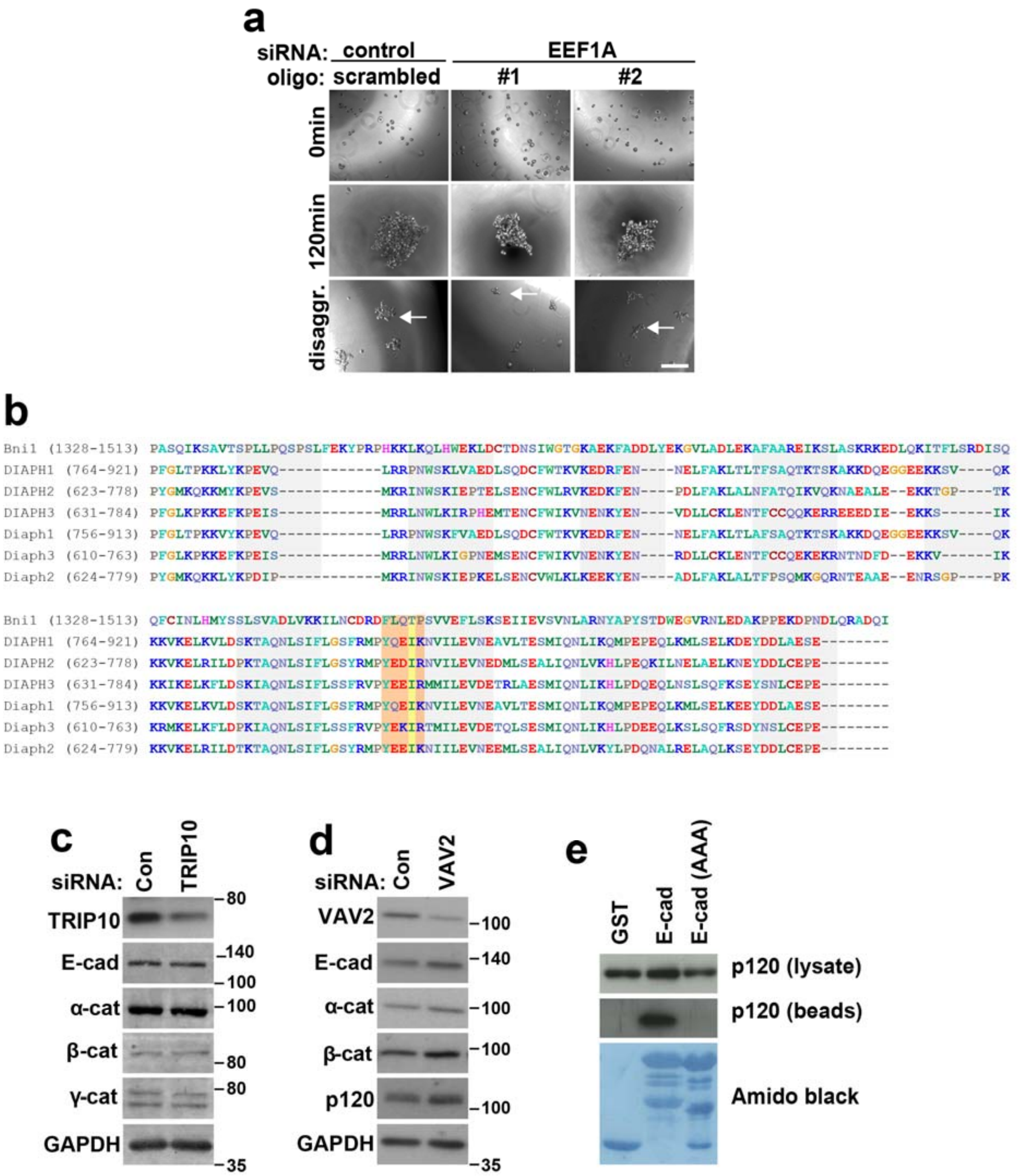

Supplementary Figure 7: Probing for EEF1A Interacting partners. **a**, Keratinocytes grown in the absence of cell-cell contacts were treated with controls or EEF1A siRNA oligos, trypsinized as single cells (0 min) and allowed to aggregate in suspension for 2 hours (120 min), followed by mechanical trituration (disaggregates; white arrows). **b**, ClustalW alignment of the EBS binding region of Bni1[1] and the relevant sequences in human (DIAPH1-3) and mouse (Diaph1-3) genes. Shaded in peach are conserved residues of the predicted EEF1A binding site that was substituted for glycine at amino acids Y713, E714, K715, and R717 in Diaph3 (mDia2). This region has a weak homology with the SEC7 domain and Y713 is phosphorylated by Src *in vitro* (A.Alberts personal communication). Residue I716 (shaded yellow) within the conserved motif was not changed. **c-d**, Total protein levels of E-cadherin and catenins after TRIP10 (c) or VAV2 (d) RNAi. GAPDH was used as loading control. Molecular weight markers are shown on the right. e, E-cadherin tail was mutated on the p120^{CTN} binding site as alanine residues (E-Cad AAA) and tested for interaction with endogenous p120^{CTN} in keratinocyte lysates. GST and GST-E-cadherin tail wild-type (E-Cad) were used as negative controls. Scale bar = 200 μM.

figure 6k

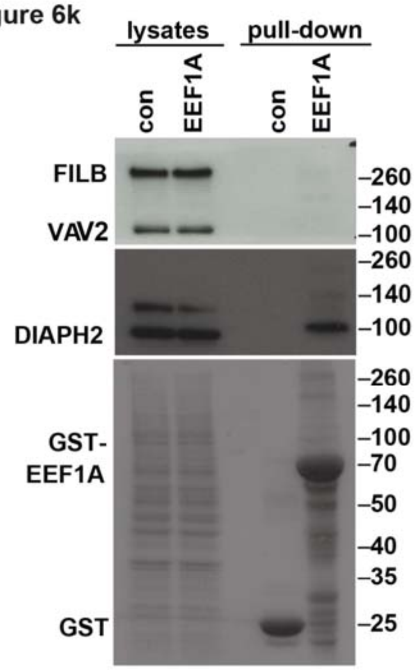

figure 6l

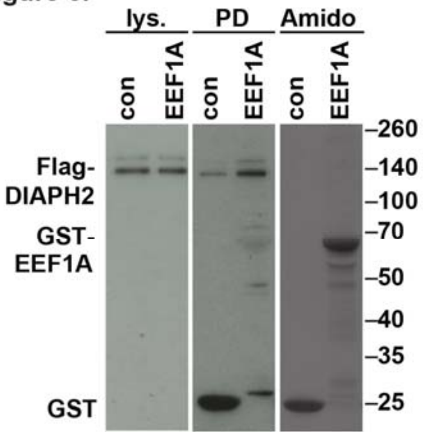

figure 6m

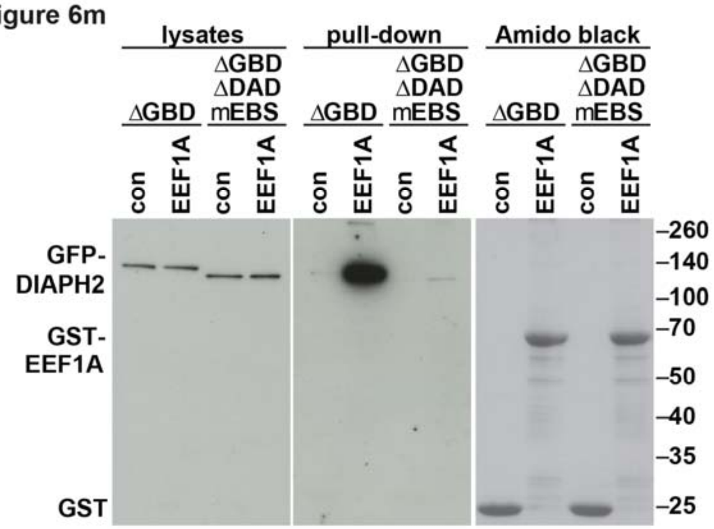

Supplementary Figure 8: Uncropped western blots of data found in Figure 6. Identification of data is written on the top left of each panel.

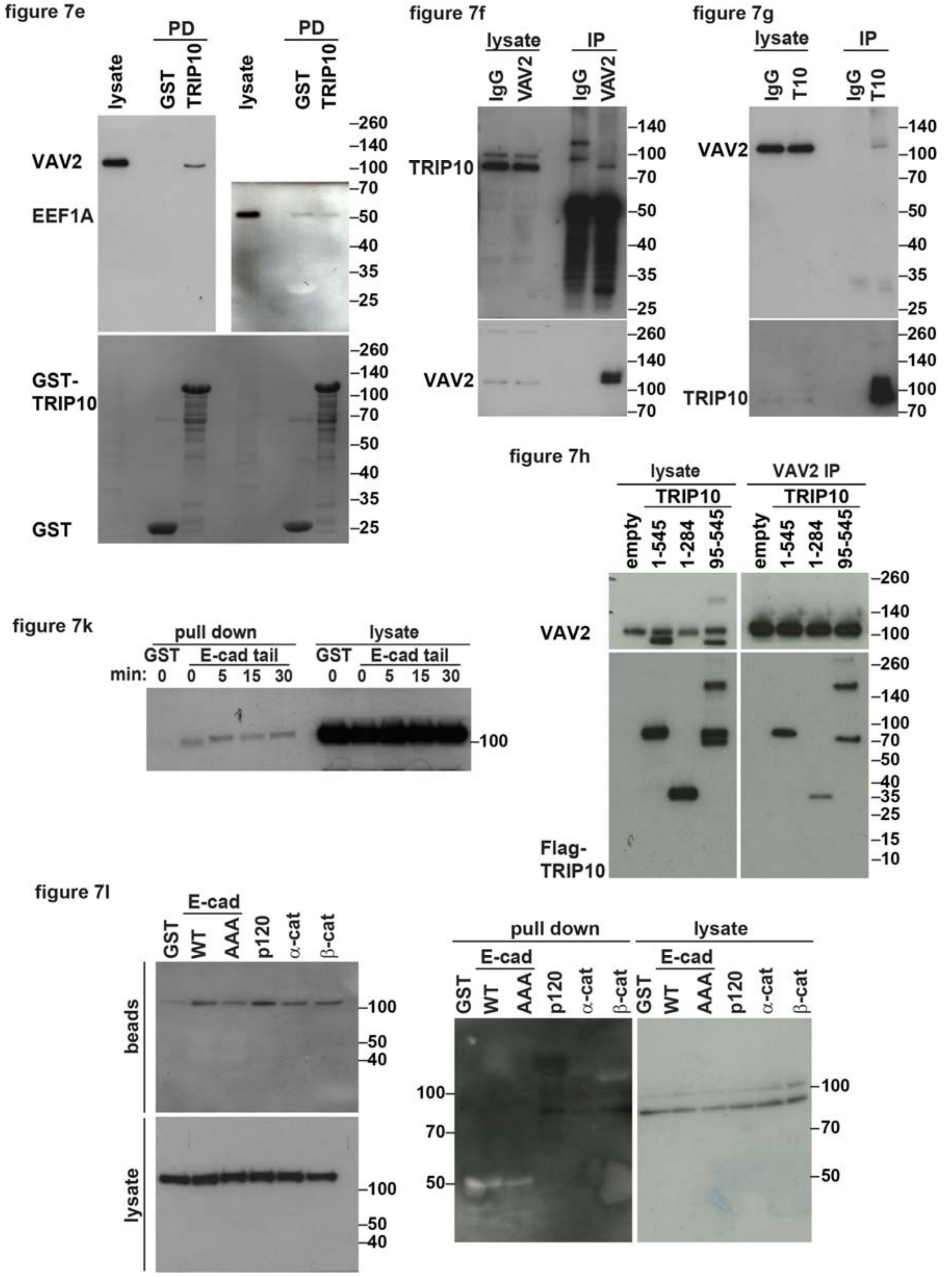

Supplementary Figure 9: Uncropped western blots of data found in Figure 7. Identification of data is written on the top left of each panel.

Supplementary Table 1: List of candidate proteins identified in the primary screen.

Number	Gene Symbol
1	ABI2
2	ACTG2
3	ACTL6
4	ACTL7B
5	ACTN3
6	ACTN4
7	ACTR1B
8	ACTRT1
9	ADD2
10	AFAP1
11	ARPC2
12	ARPC5
13	ARPC5L
14	ASPM
15	AVIL
16	AZI1
17	CALD1
18	CAP2
19	CAPZA1
20	CAPZB
21	CEACAM1
22	CEACAM5
23	CEACAM6
24	CGN
25	CIT
26	CNN2
27	CNN3
28	COBL
29	CORO1A
30	CORO2A
31	COTL1
32	CTNNA2
33	CTNNA3
34	CYFIP1
35	DCTN1
36	DIAPH2
37	DMD
38	EEF1A1
39	EHBP1
40	EHBP1L1
41	ELA1
42	ENAH
43	EPB41

Number	Gene Symbol
46	EPRS
47	EPS8
48	EZR
49	FLNA
50	FLNB
51	FLNC
52	FRMD1
53	FSCN3
54	GARNL3
55	GAS2
56	GMFB
57	GSN
58	HIP1
59	IQGAP3
60	KLHL2
61	LIMD1
62	LMOD1
63	LMOD2
64	LRCH1
65	MAP4K1
66	MAP4K3
67	MICAL3
68	MINK1
69	MTSS1
70	MYH1
71	MYH13
72	MYH14
73	MYH3
74	MYH4
75	MYH7
76	MYH7B
77	MYH8
78	MYLIP
79	MYO15A
80	MYO18B
81	MYO1D
82	MYO5A
83	MYO5B
84	MYO5C
85	MYO6
86	NAV2
87	PARVA
88	PARVB

Number	Gene Symbol
91	PLS1
92	PTK2B
93	PTPN21
94	PXK
95	PXN
96	RAPSN
97	RDX
98	SEC23A
99	SEC23B
100	SEC24A
101	SEC24B
102	SEC24C
103	SH3RF1
104	SI
105	SMC1A
106	SMTN
107	SPRED1
108	SPTBN1
109	SPTBN2
110	SPTBN4
111	SPTBN5
112	SVIL
113	SYNE1
114	SYNE2
115	TAGLN2
116	TAGLN3
117	TBCB
118	TGFB1I1
119	TLN1
120	TLN2
121	TLR5
122	TMOD1
123	TMOD2
124	TMOD3
125	TMSB10
126	TMSB4X
127	TMSB4Y
128	TNNI1
129	TNNI2
130	TNNI3
131	TNNT2
132	TNNT3
133	TPM1

Number	Gene Symbol
136	TPM4
137	TRIM3
138	TRIM45
139	TRIOBP
140	TRIP10
141	TWF1
142	UNC13A
143	USP6
144	VASP
145	VAV2
146	VAV3
147	VCL
148	VIL1
149	VILL
150	VPS39
151	VPS52
152	VTI1B
153	WAS
154	WASF1
155	WASF2
156	WIPF1

44	EPB41L1	89	PFN4	134	TPM2
45	EPB41L5	90	PLEC	135	TPM3

Supplementary Table 2: Selected candidate proteins previously identified as regulators of cadherin adhesion.

Gene	Phenotype	Reference
**ABI2**	antagonizes ENA function	2, 3
**ACTN4**	regulates actin assembly at cadherin complexes and integrity of tight junctions	4
**ADD2**	depletion attenuates cadherin contacts	5
**ARPC2**	actin organization at cadherin contacts	6, 7, 8
**CTNNA2**	α-catenin family member	
**CTNNA3**	α-catenin family member	
**CYFIP1**	depletion inhibits cell-cell junction assembly	9
**DCTN1**	ER export N-cadherin/β-catenin	10, 11
**DIAPH2**	similar to DIAPH, interaction with Abi1 at junctions is potentiated by active Rac1	6
**ENAH**	actin organization at cadherin contacts	3, 12
**EPB41L5**	regulates cadherin internalization and EMT	13
**EZR**	regulates cadherin stability and trafficking	14, 15, 16
**FLNA**	adhesion in cardiac and endothelial cells	17
**FLNB**	N-cadherin; E-cadherin apical accumulation adhesion molecules MDCK	18
**GSN**	cadherin switch; N-cadherin adhesion	19, 20
**MINK1**	kinase dead expression increases levels of cell-cell adhesion	21
**MTSS1**	stabilizes junctions; prevents cadherin contact disassembly during scattering	22, 23
**MYO6**	E-cadherin transport/stabilization	24, 25, 26
**PLEC1**	DE-cadherin remodelling	27
**PTK2B**	regulation of VE-cadherin adhesion	28, 29
**RDX**	disruption E-cadherin adhesion	30, 31
**SPTBN1**	linkage cadherin complexes to actin cytoskeleton	32, 33, 34
**TLN1**	C-terminus necessary for stability of N-cadherin in fibroblasts	35
**TRIOBP**	down-regulation E-cadherin expression	36
**TRIP10**	endocytosis of Drosophila E-cadherin	37
**VASP**	actin organization at cadherin contacts	3, 12, 38, 39, 40
**VAV2**	EGFR and VEGFR-dependent perturbation of E- and VE-cadherin contacts	41, 42
**VCL**	maintenance of cell-cell contacts, mechanosensing	25, 43, 44, 45
**WAS**	actin organization at cadherin contacts	12, 46, 47, 48, 49
**WASF1**	actin organization at cadherin contacts	50
**WASF2**	actin organization at cadherin contacts	50

Supplementary Table 3: Selected candidate proteins known to regulate epithelial morphogenesis, cell shape, polarization and differentiation.

Protein	Phenotype	Reference
**ABI2**	dendritic spine morphogenesis	2
**ACTN4**	tight junction formation	4
**ACTL7B**	hypertensive nephropathy	51
**ADD2**	bronchial epithelial cells; nephropathy	52, 53
**CEACAM1**	mammary lumen formation; desmosome organization	54, 55, 56
**CEACAM6**	blocks colonocyte differentiation	57
**CYFIP**	eye morphogenesis Drosophila	58, 59
**EBP4.1**	septate junctions Drosophila	60, 61
**EPB41L5**	epithelial cell shape; junction morphology,apical constriction	62, 63
**EPS8**	seminiferous epithelium tight junctions; apical borders of intestinal epithelial cells	64, 65, 66
**EZR**	brush borders	67, 68, 69
**FLNA**	abnormal cardiomyocites, epithelia and endothelia; perturbed adherens junctions	17
**FLNB**	impaired microvascular development	70
**MYH14**	hearing impairment	71
**MYO15A**	deafness, inner ear sterocilia formation	72, 73
**MYO5B**	microvillus inclusion disease; bile canalicular formation; epithelial polarity, lumen formation	74, 75, 76, 77
**MYO6**	hearing loss	78
**MYO7B**	chronic kidney disease	79
**PLEC1**	tracheal morphogenesis; lumen formation, epithelial integrity	27, 80
**PLS1**	terminal web assembly intestinal epithelium	81
**PTK2B**	glomerulonephritis; asthma inflamation and response	82, 83
**PXK**	systemic lupus	84
**RDX**	polarization of hepatocytes; non-syndromic hearing loss; microvillae	30, 85, 86
**SEC24B**	planar cell polarity Drosophila	87
**SPTBN2**	biogenesis lateral domain bronchial epithelia; epithelial morphology	32, 33
**TGFB1I1**	intestinal epithelia differentiation; endothelial morphology and remodelling	88, 89, 90
**TMOD1**	hexagonal geometry lens	91
**TMOD3**	epithelial morphology	92
**TRIOBP**	non-syndromic hearing loss, hair cell sterocilia	93, 94
**TRL5**	antigen processing basolateral doman; flagelling recognition	95, 96
**UNC13A**	renal injury in hyperglycemia; diabetic nephropathy	97, 98
**VASP**	Drosophila morphogenesis; airway epithelia Inflamation; endothelial barrier function	99, 100, 101, 102
**VAV2**	differentiation enterocytes; mammary acinar architecture; endothelial cell permeability	41, 42, 103
**VIL1**	assembly epithelial cell brush border	104
**WAS**	*C.elegans* epidermal morphogenesis; apical constriction Drosophila	46, 47

Materials and Methods

Surface E-cadherin assessment by ELISA:

Keratinocytes cultured in low Ca^{2+} conditions were siRNA transfected and junction formation induced by addition of calcium. Surface proteins were biotinylated with Sulfo-NHS-SS-Biotin, EZ-Link (Thermo Scientific Pierce). Cells were lysed in lysis buffer (10% Glycerol, 50mM Tris-HCl, pH7.5, 200mM NaCl, 1% (v/v) NP-40, 2mM $MgCl_2$) and protein depletion assessed by Western blot. Polystyrene microtiter plates were coated with anti-E-cadherin antibody (HECD1) over night, blocked with 5% (w/v) BSA and incubated with diluted lysates overnight. Bound protein was visualised with HRP-conjugated streptavidin and TMB substrate (BD Biosciences).

Assays to determine junction functionality:

For aggregation assays, confluents cultures of keratinocytes were trypsinised in buffer (60% versene (v/v), 0.1% trypsin 0.1mM $CaCl_2$) and re-suspended to a density of 5 X 10^4 cells/ml in standard calcium medium as described.[105] Cells were allowed to aggregate in suspension for 120 minutes and then pipetted gently to disaggregate. Remaining aggregates were imaged and their area quantified using ImageJ.

To cluster E-cadherin receptors, polystyrene beads (15µM, Polysciences) were coated with BSA or antibody against E-cadherin (HECD-1) and blocked with heat denatured BSA as described[106]. Beads were resuspended in low calcium medium and incubated with cells for 30 minutes, before cells were fixed and stained for F-actin. The percentage of attached beads that contained F-actin recruitment (enrichment, filaments or >3 discrete dots) was quantified.

To assess detergent insolubility of cadherin receptors, cells induced to form contacts for 30 minutes were pre-permeabilized with CSK buffer (10mM PIPES pH6.8, 50mM NaCl, 3mM $MgCl_2$, 300mM sucrose and 0.5% Triton X-100) for 10 minutes at room temperature prior to fixation in 3% paraformaldehyde and staining.[107]

Western blot and interaction assays:

For immunoprecipitation experiments, cultured keratinocytes were lysed (0.5% Triton X-100, 10% Glycerol, 50mM Tris-HCl pH 7.5, 150mM NaCl, 5µg/ml leupeptin, 5µg/ml pefabloc, 5µg/ml, pepstatin, 50mM phenylmethylsulfonyl fluoride, 20mM sodium flouride). After centrifugation at 8.800X*g* at 4°C for 5 minutes, the supernatants were pre-cleared using 50µl protein A/G-Sepharose 4B beads (Sigma) for two hours. Lysates were centrifuged at 2,415X at 4°C for 1 minute and the supernatant subjected to immunoprecipitation overnight with TRIP10 (3µg) or VAV2 (0.85µg) antibody. The antibody was precipitated with 50µl protein A/G-Sepharose bead slur for 2 hours at 4°C. Beads were washed three times in wash buffer (50mM Tris-HCl pH 7.5, 150mM NaCl) and SDS-PAGE sample buffer added to beads and boiled. The total volume of bead suspension was loaded.

Keratinocytes lysates prepared as above were added to immobilized GST-TRIP10 or GST-EEF1A on beads (Glutathione sepharose, GE Healthcare) for two hours at 4°C and washed as described for immunoprecipitations above. The interaction of E-cadherin tail VAV2 was performed essentially as described[108]. Following incubation, beads were incubated with lysates for 1 hour, washed in buffer with the same composition as the lysis buffer, but containing 300mM NaCl. For interaction of VAV2 and TRIP10 with cadherin tail mutant and catenins the pull down was performed in the same way, but with 150mM NaCl in the lysis and wash buffer. Washed beads were re-suspended in sample buffer and the whole sample loaded on SDS-PAGE. Protein samples were separated by SDS-PAGE, transferred onto PVDF membrane (Millipore), and immunoblotted using standard techniques.

Fluorescence recovery and analysis:

For FRAP experiments, cells were seeded onto glass bottom dishes (MatTek) and maintained in standard calcium medium. Throughout imaging cells were kept at 37°C in phenol red-free DMEM/F-12 (1:1) medium with HEPES (Gibco, Life Technologies) supplemented as described for normal keratinocyte cultures. Five pre-bleach images were acquired before photobleaching of a 14x35 pixel region at a central

part of cell-cell contacts using the 488 nm laser at 100% laser power with 50 iterations. Post-bleach imaging was performed at 4% 488 nm laser power for 192 seconds with 8 seconds intervals. Only junctions between expressing and non-expressing cells were imaged.

Actin recovery was quantified using ImageJ using a method adapted from [109]. Briefly, a region of 14x35 pixels was drawn over the bleached area and raw integrated intensity values was measured. ROI location in cells moving during the video recording was manually adjusted as necessary. To remove noise, mean intensity values of background was measured in a large area outside the cell, was normalized to the size of the bleached area and was subtracted from the raw integrated intensity values of the bleached area. To account for photo-bleaching, the corrected intensity values in the bleached area were further divided by mean cytoplasmic intensity as measured in a large region covering the cytoplasm of the transfected cell. The resulting normalised intensity values were fitted with a single exponential function in GraphPad Prism, yielding recovery plateau and recovery half-time. Only curves with R^2 values above 0.75 and reaching a plateau during the observation period were included in the analysis.

Supplementary References:

1. Umikawa M*, et al.* Interaction of Rho1p target Bni1p with F-actin-binding elongation factor 1alpha: implication in Rho1p-regulated reorganization of the actin cytoskeleton in Saccharomyces cerevisiae. *Oncogene* **16**, 2011-2016 (1998).

2. Grove M*, et al.* ABI2-deficient mice exhibit defective cell migration, aberrant dendritic spine morphogenesis, and deficits in learning and memory. *Mol Cell Biol* **24**, 10905-10922 (2004).

3. Sheffield M, Loveless T, Hardin J, Pettitt J. C. elegans Enabled exhibits novel interactions with N-WASP, Abl, and cell-cell junctions. *Curr Biol* **17**, 1791-1796 (2007).

4. Tang VW, Brieher WM. Alpha-actinin-4/FSGS1 is required for Arp2/3-dependent actin assembly at the adherens junction. *J Cell Biol* **196**, 115-130 (2012).

5. Naydenov NG, Ivanov AI. Adducins regulate remodeling of apical junctions in human epithelial cells. *Mol Biol Cell* **21**, 3506-3517 (2010).

6. Ryu JR, Echarri A, Li R, Pendergast AM. Regulation of cell-cell adhesion by Abi/Diaphanous complexes. *Mol Cell Biol* **29**, 1735-1748 (2009).

7. Verma S*, et al.* Arp2/3 activity is necessary for efficient formation of E-cadherin adhesive contacts. *J Biol Chem* **279**, 34062-34070 (2004).

8. Kovacs EM, Goodwin M, Ali RG, Paterson AD, Yap AS. Cadherin-directed actin assembly: E-cadherin physically associates with the Arp2/3 complex to direct actin assembly in nascent adhesive contacts. *Curr Biol* **12**, 379-382 (2002).

9. Silva JM*, et al.* Cyfip1 is a putative invasion suppressor in epithelial cancers. *Cell* **137**, 1047-1061 (2009).

10. Lien WH, Gelfand VI, Vasioukhin V. Alpha-E-catenin binds to dynamitin and regulates dynactin-mediated intracellular traffic. *J Cell Biol* **183**, 989-997 (2008).

11. Nakamura T*, et al.* The PX-RICS-14-3-3zeta/theta complex couples N-cadherin-beta-catenin with dynein-dynactin to mediate its export from the endoplasmic reticulum. *J Biol Chem* **285**, 16145-16154 (2010).

12. Vasioukhin V, Bauer C, Yin M, Fuchs E. Directed actin polymerization is the driving force for epithelial cell-cell adhesion. *Cell* **100**, 209-219 (2000).

13. Hirano M, Hashimoto S, Yonemura S, Sabe H, Aizawa S. EPB41L5 functions to post-transcriptionally regulate cadherin and integrin during epithelial-mesenchymal transition. *J Cell Biol*, jcb.200712086 (2008).

14. Hiscox S, Jiang W, G,. Ezrin regulates cell-cell and cell-matrix adhesion, a possible role with E-cadherin/b-catenin. *J Cell Sci* **112**, 3081-3090 (1999).

15. Pujuguet P, Del Maestro L, Gautreau A, Louvard D, Arpin M. Ezrin regulates E-Cadherin-dependent adherens junction assembly through Rac1 activation. *Mol Biol Cell* **14**, 2181-2191 (2003).

16. van Furden D, Johnson K, Segbert C, Bossinger O. The *C. elegans* ezrin-radixin-moesin protein ERM-1 is necessary for apical junction remodelling and tubulogenesis in the intestine. *Dev Biol* **272**, 262-276 (2004).

17. Feng Y*, et al.* Filamin A (FLNA) is required for cell-cell contact in vascular development and cardiac morphogenesis. *Proc Natl Acad Sci USA* **103**, 19836-19841 (2006).

18. Wakamatsu Y, Sakai D, Suzuki T, Osumi N. FilaminB is required for the directed localization of cell-cell adhesion molecules in embryonic epithelial development. *Develop Dyn* **240**, 149-161 (2011).

19. Tanaka H*, et al.* siRNA gelsolin knockdown induces epithelial-mesenchymal transition with a cadherin switch in human mammary epithelial cells. *Int J Cancer* **118**, 1680-1691 (2006).

20. El Sayegh TY*, et al.* Phosphatidylinositol-4,5 bisphosphate produced by PIP5KIgamma regulates gelsolin, actin assembly, and adhesion strength of N-cadherin junctions. *Mol Biol Cell* **18**, 3026-3038 (2007).

21. Hu Y*, et al.* Identification and functional characterization of a novel human misshapen/Nck interacting kinase-related kinase, hMINK beta. *J Biol Chem* **279**, 54387-54397 (2004).

22. Dawson JC, Bruche S, Spence HJ, Braga VMM, Machesky LM. Mtss1 promotes cell-cell junction assembly and stability through the small GTPase Rac1. *PLoS One* **7**, e31141 (2012).

23. Saarikangas J*, et al.* Missing-in-metastasis MIM/MTSS1 promotes actin assembly at intercellular junctions and is required for integrity of kidney epithelia. *J Cell Sci* **124**, 1245-1255 (2011).

24. Geisbrecht ER, Montell DJ. Myosin VI is required for E-cadherin-mediated border cell migration. *Nature Cell Biol* **4**, 616-620 (2002).

25. Maddugoda MP, Crampton MS, Shewan AM, Yap AS. Myosin VI and vinculin cooperate during the morphogenesis of cadherin cell cell contacts in mammalian epithelial cells. *J Cell Biol* **178**, 529-540 (2007).

26. Millo H, Leaper K, Lazou V, Bownes M. Myosin VI plays a role in cell-cell adhesion during epithelial morphogenesis. *Mechan Dev* **121**, 1335-1351 (2004).

27. Lee S, Kolodziej PA. The plakin Short Stop and the RhoA GTPase are required for E-cadherin-dependent apical surface remodeling during tracheal tube fusion. *Development* **129**, 1509-1520 (2002).

28. Cain RJ, Vanhaesebroeck B, Ridley AJ. The PI3K p110alpha isoform regulates endothelial adherens junctions via Pyk2 and Rac1. *J Cell Biol* **188**, 863-876 (2010).

29. van Buul JD, Anthony EC, Fernandez-Borja M, Burridge K, Hordijk PL. Proline-rich tyrosine kinase 2 (Pyk2) mediates vascular endothelial-cadherin-based cell-cell adhesion by regulating beta-catenin tyrosine phosphorylation. *J Biol Chem* **280**, 21129-21136 (2005).

30. Takeuchi K, *et al.* Perturbation of cell adhesion and microvilli formation by antisense oligonucleotides to ERM family members. *J Cell Biol* **125**, 1371-1384 (1994).

31. Valderrama F, Thevapala S, Ridley AJ. Radixin regulates cell migration and cell-cell adhesion through Rac1. *J Cell Sci*, (2012).

32. Hu RJ, Moorthy S, Bennett V. Expression of functional domains of beta G-spectrin disrupts epithelial morphology in cultured cells. *J Cell Biol* **128**, 1069-1080 (1995).

33. Kizhatil K, Davis JQ, Davis L, Hoffman J, Hogan BLM, Bennett V. Ankyrin-G is a molecular partner of E-cadherin in epithelial cells and early embryos. *J Biol Chem* **282**, 26552-26561 (2007).

34. Pradhan D, Lombardo CR, Roe S, Rimm DL, Morrow JS. alpha -catenin binds directly to spectrin and facilitates spectrin-membrane assembly in vivo. *J Biol Chem* **276**, 4175-4181 (2001).

35. Zhang F, Saha S, Kashina A. Arginylation-dependent regulation of a proteolytic product of talin is essential for cell-cell adhesion. *J Cell Biol* **197**, 819-836 (2012).

36. Yano T, *et al.* Tara up-regulates E-cadherin transcription by binding to the Trio RhoGEF and inhibiting Rac signaling. *J Cell Biol* **193**, 319-332 (2011).

37. Leibfried A, Fricke R, Morgan MJ, Bogdan S, Bellaiche Y. Drosophila Cip4 and WASp define a branch of the Cdc42-Par6-aPKC pathway regulating E-cadherin endocytosis. *Curr Biol* **18**, 1639-1648 (2008).

38. Hansen MDH, Beckerle MC. Opposing roles of zyxin/LPP ACTA repeats and the LIM domain region in cell-cell adhesion. *J Biol Chem* **281**, 16178-16188 (2006).

39. Kris AS, Kamm RD, Sieminski AL. VASP involvement in force-mediated adherens junction strengthening. *Biochem Biophys Res Comm* **375**, 134-138 (2008).

40. Scott JA, Shewan AM, den Elzen NR, Loureiro JJ, Gertler FB, Yap AS. Ena/VASP proteins can regulate distinct modes of actin organization at cadherin-adhesive contacts. *Mol Biol Cell* **17**, 1085-1095 (2006).

41. Duan L, *et al.* Distinct roles for Rho versus Rac/Cdc42 GTPases downstream of Vav2 in regulating mammary epithelial acinar architecture. *J Biol Chem* **285**, 1555-1568 (2010).

42. Gavard J, Gutkind JS. VEGF controls endothelial-cell permeability by promoting the b-arrestin-dependent endocytosis of VE-cadherin. *Nature Cell Biol* **8**, 1223-1234 (2006).

43. le Duc Q, *et al.* Vinculin potentiates E-cadherin mechanosensing and is recruited to actin-anchored sites within adherens junctions in a myosin II-dependent manner. *J Cell Biol* **189**, 1107-1115 (2010).

44. Palovuori R, Eskelinen S. Role of vinculin in the maintenance of cell-cell contacts in kidney epithelial MDBK cells. *Eur J Cell Biol* **79**, 961-974 (2000).

45. Peng X, Cuff LE, Lawton CD, Demali KA. Vinculin regulates cell-surface E-cadherin expression by binding to b-catenin. *J Cell Sci* **123**, 567-577 (2010).

46. Bertet C, Rauzi M, Lecuit T. Repression of Wasp by JAK/STAT signalling inhibits medial actomyosin network assembly and apical cell constriction in intercalating epithelial cells. *Development* **136**, 4199-4212 (2009).

47. Giuliani C, *et al.* Requirements for F-BAR proteins TOCA-1 and TOCA-2 in actin dynamics and membrane trafficking during Caenorhabditis elegans oocyte growth and embryonic epidermal morphogenesis. *PLoS Genet* **5**, e1000675 (2009).

48. Otani T, Ichii T, Aono S, Takeichi M. Cdc42 GEF Tuba regulates the junctional configuration of simple epithelial cells. *J Cell Biol* **175**, 135-146 (2006).

49. Kovacs EM*, et al.* N-WASP regulates the epithelial junctional actin cytoskeleton through a non-canonical post-nucleation pathway. *Nature Cell Biol* **13**, 934-943 (2011).

50. Yamazaki D, Oikawa T, Takenawa T. Rac-WAVE-mediated actin reorganization is required for organization and maintenance of cell-cell adhesion. *J Cell Sci* **120**, 86-100 (2007).

51. Chung KW*, et al.* African American hypertensive nephropathy maps to a new locus on chromosome 9q31-q32. *Am J Hum Gen* **73**, 420-429 (2003).

52. Abdi KM, Bennett V. Adducin promotes micrometer-scale organization of beta2-spectrin in lateral membranes of bronchial epithelial cells. *Mol Biol Cell* **19**, 536-545 (2008).

53. Ferrandi M*, et al.* alpha- and beta-Adducin polymorphisms affect podocyte proteins and proteinuria in rodents and decline of renal function in human IgA nephropathy. *J Mol Med* **88**, 203-217 (2010).

54. Chen CJ, Kirshner J, Sherman MA, Hu W, Nguyen T, Shively JE. Mutation analysis of the short cytoplasmic domain of the cell-cell adhesion molecule CEACAM1 identifies residues that orchestrate actin binding and lumen formation. *J Biol Chem* **282**, 5749-5760 (2007).

55. Yokoyama S, Chen CJ, Nguyen T, Shively JE. Role of CEACAM1 isoforms in an in vivo model of mammary morphogenesis: mutational analysis of the cytoplasmic domain of CEACAM1-4S reveals key residues involved in lumen formation. *Oncogene* **26**, 7637-7646 (2007).

56. Sundberg U, Beauchemin N, Obrink B. The cytoplasmic domain of CEACAM1-L controls its lateral localization and the organization of desmosomes in polarized epithelial cells. *J Cell Sci* **117**, 1091-1104 (2004).

57. Ilantzis C, DeMarte L, Screaton RA, Stanners CP. Deregulated expression of the human tumor marker CEA and CEA family member CEACAM6 disrupts tissue architecture and blocks colonocyte differentiation. *Neoplasia* **4**, 151-163 (2002).

58. Galy A*, et al.* CYFIP dependent actin remodeling controls specific aspects of Drosophila eye morphogenesis. *Dev Biol* **359**, 37-46 (2011).

59. Toret CP, D'Ambrosio MV, Vale RD, Simon MA, Nelson WJ. A genome-wide screen identifies conserved protein hubs required for cadherin-mediated cell-cell adhesion. *J Cell Biol* **204**, 265-279 (2014).

60. Fehon RG, Dawson IA, Artavanis-Tsakonas S. A Drosophila homologue of membrane-skeleton protein 4.1 is associated with septate junctions and is encoded by the coracle gene. *Development* **120**, 545-557 (1994).

61. Lamb RS, Ward RE, Schweizer L, Fehon RG. Drosophila coracle, a member of the protein 4.1 superfamily, has essential structural functions in the septate junctions and developmental functions in embryonic and adult epithelial cells. *Mol Biol Cell* **9**, 3505-3519 (1998).

62. Nakajima H, Tanoue T. Epithelial cell shape is regulated by Lulu proteins via myosin-II. *J Cell Sci* **123**, 555-566 (2010).

63. Gosens I*, et al.* FERM protein EPB41L5 is a novel member of the mammalian CRB-MPP5 polarity complex. *Exp Cell Res* **313**, 3959-3970 (2007).

64. Croce A*, et al.* A novel actin barbed-end-capping activity in EPS-8 regulates apical morphogenesis in intestinal cells of Caenorhabditis elegans. *Nature Cell Biol* **6**, 1173-1179 (2004).

65. Lie PP, Mruk DD, Lee WM, Cheng CY. Epidermal growth factor receptor pathway substrate 8 (Eps8) is a novel regulator of cell adhesion and the blood-testis barrier integrity in the seminiferous epithelium. *FASEB J* **23**, 2555-2567 (2009).

66. Zwaenepoel I*, et al.* Ezrin regulates microvillus morphogenesis by promoting distinct activities of Eps8 proteins. *Mol Cell Biol* **23**, 1080-1094 (2012).

67. ten Klooster JP*, et al.* Mst4 and Ezrin induce brush borders downstream of the Lkb1/Strad/Mo25 polarization complex. *Dev Cell* **16**, 551-562 (2009).

68. Saotome I, Curto M, McClatchey AI. Ezrin is essential for epithelial organization and villus morphogenesis in the developing intestine. *Dev Cell* **6**, 855-864 (2004).

69. Casaletto JB, Saotome I, Curto M, McClatchey AI. Ezrin-mediated apical integrity is required for intestinal homeostasis. *Proc Natl Acad Sci USA* **108**, 11924-11929 (2011).

70. Zhou X*, et al.* Filamin B deficiency in mice results in skeletal malformations and impaired microvascular development. *Proc Natl Acad Sci USA* **104**, 3919-3924 (2007).

71. Donaudy F*, et al.* Nonmuscle myosin heavy-chain gene MYH14 is expressed in cochlea and mutated in patients affected by autosomal dominant hearing impairment (DFNA4). *Am J Hum Gen* **74**, 770-776 (2004).

72. Belyantseva IA*, et al.* Myosin-XVa is required for tip localization of whirlin and differential elongation of hair-cell stereocilia. *Nature Cell Biol* **7**, 148-156 (2005).

73. Belyantseva IA, Boger ET, Friedman TB. Myosin XVa localizes to the tips of inner ear sensory cell stereocilia and is essential for staircase formation of the hair bundle. *Proc Natl Acad Sci USA* **100**, 13958-13963 (2003).

74. Muller T*, et al.* MYO5B mutations cause microvillus inclusion disease and disrupt epithelial cell polarity. *Nature Genet* **40**, 1163-1165 (2008).

75. Roland JT, Bryant DM, Datta A, Itzen A, Mostov KE, Goldenring JR. Rab GTPase-Myo5B complexes control membrane recycling and epithelial polarization. *Proc Natl Acad Sci USA* **108**, 2789-2794 (2011).

76. Ruemmele FM*, et al.* Loss-of-function of MYO5B is the main cause of microvillus inclusion disease: 15 novel mutations and a CaCo-2 RNAi cell model. *Human Mutat* **31**, 544-551 (2010).

77. Wakabayashi Y, Dutt P, Lippincott-Schwartz J, Arias IM. Rab11a and myosin Vb are required for bile canalicular formation in WIF-B9 cells. *Proc Natl Acad Sci USA* **102**, 15087-15092 (2005).

78. Sanggaard KM*, et al.* A novel nonsense mutation in MYO6 is associated with progressive nonsyndromic hearing loss in a Danish DFNA22 family. *Am J Med Gen* **146A**, 1017-1025 (2008).

79. Yoshida T*, et al.* Association of gene polymorphisms with chronic kidney disease in Japanese individuals. *Int J Mol Med* **24**, 539-547 (2009).

80. Roper K, Brown NH. Maintaining epithelial integrity: a function for gigantic spectraplakin isoforms in adherens junctions. *J Cell Biol* **162**, 1305-1315 (2003).

81. Grimm-Gunter EM*, et al.* Plastin 1 binds to keratin and is required for terminal web assembly in the intestinal epithelium. *Mol Biol Cell* **20**, 2549-2562 (2009).

82. Duan Y, Learoyd J, Meliton AY, Clay BS, Leff AR, Zhu X. Inhibition of Pyk2 blocks airway inflammation and hyperresponsiveness in a mouse model of asthma. *Am J Resp Cell Mol Biol* **42**, 491-497 (2010).

83. Takagi C*, et al.* Increased expression of cell adhesion kinase beta in human and rat crescentic glomerulonephritis. *Am J Kidney Dis* **39**, 174-182 (2002).

84. Harley JB*, et al.* Genome-wide association scan in women with systemic lupus erythematosus identifies susceptibility variants in ITGAM, PXK, KIAA1542 and other loci. *Nature Genet* **40**, 204-210 (2008).

85. Suda J, Zhu L, Karvar S. Phosphorylation of radixin regulates cell polarity and Mrp-2 distribution in hepatocytes. *Am J Physiol Cell Physiol* **300**, C416-424 (2011).

86. Khan SY*, et al.* Mutations of the RDX gene cause nonsyndromic hearing loss at the DFNB24 locus. *Hum Mutat* **28**, 417-423 (2007).

87. Wansleeben C, Feitsma H, Montcouquiol M, Kroon C, Cuppen E, Meijlink F. Planar cell polarity defects and defective Vangl2 trafficking in mutants for the COPII gene Sec24b. *Development* **137**, 1067-1073 (2010).

88. Drori S*, et al.* Hic-5 regulates an epithelial program mediated by PPARgamma. *Genes Dev* **19**, 362-375 (2005).

89. Komorowsky C, Samarin J, Rehm M, Guidolin D, Goppelt-Struebe M. Hic-5 as a regulator of endothelial cell morphology and connective tissue growth factor gene expression. *Journal of Molecular Medicine (Berl)* **88**, 623-631 (2010).

90. Kim-Kaneyama JR*, et al.* Hic-5 deficiency enhances mechanosensitive apoptosis and modulates vascular remodeling. *J Mol Cell Cardiol* **50**, 77-86 (2011).

91. Nowak RB, Fischer RS, Zoltoski RK, Kuszak JR, Fowler VM. Tropomodulin1 is required for membrane skeleton organization and hexagonal geometry of fiber cells in the mouse lens. *J Cell Biol* **186**, 915-928 (2009).

92. Weber KL, Fischer RS, Fowler VM. Tmod3 regulates polarized epithelial cell morphology. *J Cell Sci* **120**, 3625-3632 (2007).

93. Shahin H*, et al.* Mutations in a novel isoform of TRIOBP that encodes a filamentous-actin binding protein are responsible for DFNB28 recessive nonsyndromic hearing loss. *Am J Hum Gen* **78**, 144-152 (2006).

94. Kitajiri S*, et al.* Actin-bundling protein TRIOBP forms resilient rootlets of hair cell stereocilia essential for hearing. *Cell* **141**, 786-798 (2010).

95. Andersen-Nissen E, Smith KD, Bonneau R, Strong RK, Aderem A. A conserved surface on Toll-like receptor 5 recognizes bacterial flagellin. *J Exp Med* **204**, 393-403 (2007).

96. Demirci FY*, et al.* Association study of Toll-like receptor 5 (TLR5) and Toll-like receptor 9 (TLR9) polymorphisms in systemic lupus erythematosus. *J Rheumatol* **34**, 1708-1711 (2007).

97. Song Y, Ailenberg M, Silverman M. Cloning of a novel gene in the human kidney homologous to rat munc13s: its potential role in diabetic nephropathy. *Kidney Int* **53**, 1689-1695 (1998).

98. Song Y, Ailenberg M, Silverman M. Human munc13 is a diacylglycerol receptor that induces apoptosis and may contribute to renal cell injury in hyperglycemia. *Mol Biol Cell* **10**, 1609-1619 (1999).

99. Gates J*, et al.* Enabled plays key roles in embryonic epithelial morphogenesis in Drosophila. *Development* **134**, 2027-2039 (2007).

100. Hastie AT, *et al.* Alterations in vasodilator-stimulated phosphoprotein (VASP) phosphorylation: associations with asthmatic phenotype, airway inflammation and beta2-agonist use. *Resp Res* **7**, 25 (2006).

101. Schlegel N, Burger S, Golenhofen N, Walter U, Drenckhahn D, Waschke J. The role of VASP in regulation of cAMP- and Rac 1-mediated endothelial barrier stabilization. *Am J Physiol Cell Physiol* **294**, C178-C188 (2008).

102. Furman C, *et al.* Ena/VASP is required for endothelial barrier function in vivo. *J Cell Biol* **179**, 761-775 (2007).

103. Liu JY, Seno H, Miletic AV, Mills JC, Swat W, Stappenbeck TS. Vav proteins are necessary for correct differentiation of mouse cecal and colonic enterocytes. *J Cell Sci* **122**, 324-334 (2009).

104. George SP, Wang Y, Mathew S, Srinivasan K, Khurana S. Dimerization and actin-bundling properties of villin and its role in the assembly of epithelial cell brush borders. *J Biol Chem* **282**, 26528-26541 (2007).

105. Nola S, *et al.* Ajuba is required for Rac activation and maintenance of E-cadherin adhesion. *J Cell Biol* **195**, 855-871 (2011).

106. Braga VMM, Machesky LM, Hall A, Hotchin NA. The small GTPases Rho and Rac are required for the establishment of cadherin-dependent cell-cell contacts. *J Cell Biol* **137**, 1421-1431 (1997).

107. Braga VMM, Hodivala KJ, Watt FM. Calcium-induced changes in distribution and solubility of cadherins and their associated cytoplasmic proteins in human keratinocytes. *Cell Adh Comm* **3**, 201-215 (1995).

108. Erasmus JC, Welsh NJ, Braga VMM. Cooperation of distinct Rac-dependent pathways to stabilise E-cadherin adhesion. *Cell Signal* **27**, 1905-1913 (2015).

109. Keller D, *et al.* Mechanisms of HsSAS-6 assembly promoting centriole formation in human cells. *J Cell Biol* **204**, 697-712 (2014).
